# Transcriptomic Drivers of Differentiation, Maturation, and Polyploidy in Human Extravillous Trophoblast

**DOI:** 10.3389/fcell.2021.702046

**Published:** 2021-09-03

**Authors:** Robert Morey, Omar Farah, Sampada Kallol, Daniela F. Requena, Morgan Meads, Matteo Moretto-Zita, Francesca Soncin, Louise C. Laurent, Mana M. Parast

**Affiliations:** ^1^Department of Pathology, University of California, San Diego, La Jolla, CA, United States; ^2^Department of Obstetrics, Gynecology, and Reproductive Sciences, Division of Maternal-Fetal Medicine, University of California, San Diego, La Jolla, CA, United States; ^3^Sanford Consortium for Regenerative Medicine, University of California, San Diego, La Jolla, CA, United States

**Keywords:** extravillous trophoblast, placenta, polyploid, senescence, cytotrophoblast

## Abstract

During pregnancy, conceptus-derived extravillous trophoblast (EVT) invades the endomyometrium, anchors the placenta to the maternal uterus, and remodels the spiral arteries in order to establish maternal blood supply to the fetoplacental unit. Recent reports have described early gestation EVT as polyploid and senescent. Here, we extend these reports by performing comprehensive profiling of both the genomic organization and transcriptome of first trimester and term EVT. We define pathways and gene regulatory networks involved in both initial differentiation and maturation of this important trophoblast lineage at the maternal–fetal interface. Our results suggest that like first trimester EVT, term EVT undergoes senescence and endoreduplication, is primarily tetraploid, and lacks high rates of copy number variations. Additionally, we have highlighted senescence and polyploidy-related genes, pathways, networks, and transcription factors that appeared to be important in normal EVT differentiation and maturation and validated a key role for the unfolded protein response in this context.

## Introduction

The human placenta is essential for successful pregnancy and unique in its transitory nature. It performs a multitude of functions, including gas and nutrient exchange, synthesis of pregnancy-specific signaling molecules, and induction of maternal immunological tolerance. The placenta is also unique in its invasive nature. Early in development, the placenta displays tumor-like properties as one of its component cell types, the extravillous trophoblast (EVT), aggressively invades the endomyometrium of the maternal uterus, and remodels the spiral arteries (Pijnenborg et al., 1980). EVTs arise from the proliferative epithelial stem cells of the placenta, the cytotrophoblast (CTB), and exit the cell cycle as they differentiate and invade. EVTs share many of the molecular hallmarks of cancer cells (Ferretti et al., 2007), one of which is the frequent occurrence of structural genomic rearrangements and aneuploidy (Sansregret and Swanton, 2017). Trophoblast giant cells (TGCs), the mouse equivalent to EVTs, are known to be highly polyploid (Barlow and Sherman, 1972; Zybina et al., 2011), meaning that they possess more than two sets of chromosomes and undergo endoreduplication, a process by which cells undergo DNA replication in the absence of subsequent cell division (Fox and Duronio, 2013). Further studies have shown that TGCs harbor consistent regions of copy number variation (CNV) that may function as an important mode of genome regulation (Hannibal et al., 2014; Hannibal and Baker, 2016). Compared with rodents, there are only a small number of previous studies examining human trophoblast polyploidy or genomic CNVs (Zybina et al., 2002, 2004; Weier et al., 2005; Meinhardt et al., 2015). One such study in the human placenta showed an enrichment of CNVs, suggesting that, as in the mouse, the human placenta contains an atypical genome architecture that is important for the normal function of the organ (Kasak et al., 2015). Recently, however, another study focused on invasive EVT in first trimester human placenta and reported that, unlike mouse TGCs, these cells did not contain CNVs but were predominantly tetraploid, and potentially undergo senescence and endoreduplication (Velicky et al., 2018). However, to date, similar analysis of term EVT has not been done.

At the same time, several groups, including ours, have characterized the transcriptome of human first trimester EVT using microarray-based profiling (Apps et al., 2011; Telugu et al., 2013; Tilburgs et al., 2015; Wakeland et al., 2017). These studies have shown that, compared with their CTB precursors, EVTs down-regulate pathways involving cell cycle, oxidative phosphorylation, p53, and fatty acid metabolism while upregulating those involved in immune response, hypoxia- and hypoxia-inducible factor, mTOR signaling, and epithelial–mesenchymal transition (EMT), of which the latter has been most extensively studied during EVT differentiation (Apps et al., 2011; Telugu et al., 2013; Dasilva-Arnold et al., 2015; Tilburgs et al., 2015; Davies et al., 2016; Wakeland et al., 2017). Nevertheless, there is a paucity of data, both on the gene expression profile of term EVT and on gene regulatory networks associated with EVT differentiation, maturation, and polyploidy.

Here, we aimed to extend the recent studies discussed above by performing comprehensive profiling of the genomic organization and transcriptome of first trimester and term EVT. To this end, we have used single-cell and bulk whole genome sequencing (WGS) data, along with SNP genotyping, to investigate CNVs in first trimester and term EVT, compared with CTB and umbilical cord mesenchymal stem cells. We also used RNA sequencing to characterize the transcriptomes of both first trimester and term CTB and EVT, in order to identify pathways involved in EVT differentiation and maturation, as well as those that play a role in establishment of polyploidy in these cells. We also use the newly developed method of human trophoblast stem cell (hTSC) derivation and differentiation (Okae et al., 2018) to evaluate development of polyploidy in *in vitro*-differentiated EVT, and to validate the unfolded protein response (UPR) as a newly identified pathway involved in EVT function. Finally, we also analyze our RNA-seq data to identify the TF networks involved in normal EVT formation and function.

## Materials and Methods

### Placenta Samples, Cell Isolation, and EVT Differentiation

Human placental tissues were collected under a UCSD Human Research Protections Program Committee Institutional Review Board-approved protocol; all patients provided informed consent for collection and use of these tissues. Cells were isolated from a total of 46 normal placentae, 27 first trimester and 19 term (Supplementary Table 1). Gestational age (GA) is stated in weeks and days since the last menstrual period. For first trimester placentae, “normal” refers to elective termination of pregnancy in the absence of structural fetal abnormalities; for third trimester placentae (term), “normal” is defined by a non-hypertensive, non-diabetic singleton pregnancy, where the placenta is normally grown and shows no gross or histological abnormalities.

Isolation of CTB and EVT from 20 term placentae (37–41 weeks GA) was performed as previously described in Li et al. (2013). Briefly, the placentae were obtained immediately after C-section and placed on ice. Tissue from the basal plate (for EVT) and chorionic portion (for CTB) was dissected and minced, rinsed in 1 × PBS (Corning), and digested for 20 min in 1 × Ca/Mg-free Hanks’ Balanced Salt Solution (HBSS; Gibco), 1 × trypsin (Gibco), collagenase, and DNase (Roche) three times, discarding the supernatant after each digestion. A Percoll^®^ gradient (Sigma-Aldrich) centrifugation separation was then performed. Cells were then subjected to positive selection using magnetic activated cell sorting (MACS; Miltenyi Biotec) and a PE-conjugated antibody against HLA-G (EXBIO MEM-G/9). The bound fraction was collected and tested for purity using flow cytometry. EVT preparations that contained greater than 90% HLA-G^+^ cells were considered as adequate and used in downstream experiments. The unbound fraction was collected, and CTB was selected using APC-conjugated antibody against EGFR (Biolegend #352906) and tested for purity using flow cytometry. CTB preparations yielding greater than 90% EGFR positivity were considered adequate and used in downstream experiments. Isolation of primary first trimester (9–14 weeks GA) trophoblast cells from 27 placenta samples was performed as previously described in Wakeland et al. (2017) and Soncin et al. (2018). Briefly, chorionic villi were minced, washed in HBSS (Gibco), and digested three times with DNase I (Roche), and trypsin (Gibco). The cells were then pelleted, separated on a Percoll gradient (Sigma-Aldrich) and subjected to sequential MACS selection similar to the term placental samples.

Human trophoblast stem cell lines were derived from early first trimester (6–7 weeks GA) placentae as previously described by Okae et al. (2018). Briefly, placental villi were minced, enzymatically digested, and then filtered. After Percoll^®^ separation, the cells in the trophoblast fraction were MACS-purified with a PE-conjugated anti-ITGA6 antibody (Biolegend #313612; cell line 1,049) or an APC-conjugated anti-EGFR antibody (Biolegend #352906; cell line 1,048). The 1270C hTSC line was derived directly from the trophoblast fraction (after Percoll gradient) of the chorionic side of the placental tissue (manually separated from the basal side). Cells were then plated on collagen IV-coated 6-well plates for at least 1 h on TS media as described previously in Okae et al. (2018). Cells were first grown in modified basal media (advanced DMEM/F12, N2/B27 supplements, 2 mM glutamine, 10 μM 1-thioglycerol, 0.05% BSA, and 1% KSR). The media was then changed to modified complete media (basal media with the addition of 2 μM CHIR99021, 500 nM A83-01, 1 μM SB431542, 5 μM Y-27632, 0.8 mM valproic acid sodium, 100 ng/ml FGF2, 50 ng/ml EGF, 20 ng/ml Noggin, and 50 ng/ml HGF) and grown to 80% confluency. Cells were passaged using TrypLE incubated for 15 min at 37°C. To characterize the trophoblast stem cell identity of these cells, their transcriptome was compared with that of cells derived by Okae et al. (2018), as well as to primary CTB and EVT; our TSCs were found to cluster with both the embryo- and placenta-derived Okae TSC (Supplementary Figure 4). EVT differentiation was performed by plating 0.75 × 10^5^ cells in a 6-well plate precoated with 20 μg/ml fibronectin using the EVT differentiation media described in Okae et al. (2018; DMEM/F12 supplemented with 0.1 mM 2-mercaptoethanol, 0.3% BSA, 1% ITS-X supplement, 100 ng/ml NRG1, 7.5 μM A83-01, 2.5 μM Y27632, 2% Matrigel, and 4% KnockOut Serum Replacement). On day 3, the medium was replaced with EVT medium lacking NRG1, and Matrigel was added to a final concentration of 0.5%. On day 5, the cells reached 80% confluency and were dissociated using TrypLE for 13 min at 37°C. The cells were assessed for differentiation efficiency by flow cytometric analysis using antibodies against HLA-G (EXBIO MEM-G/9) and EGFR (Biolegend #352906). For experiments evaluating the UPR pathway during EVT differentiation, the media was supplemented with 30 μM 4u8C (Sigma-Aldrich) or equivalent (vol/vol) amount of DMSO carrier.

Human umbilical cord (UC) was collected aseptically under a protocol approved by the Human Research Protections Program Committee of the UCSD Institutional Review Board (IRB number: 181917X). All patients provided informed consent for collection and use of these tissues, and all experiments were performed within guidelines and regulations set forth by the IRB. Umbilical cord mesenchymal stem cells (UC-MSCs) were derived from minced UC tissue per a published protocol (Ishige et al., 2009; Yang et al., 2014). UC samples were collected and processed within 24 h of delivery. Briefly, UCs were minced, washed to remove blood, and then cultured in basal medium [aMEM with nucleosides (ThermoFisher), containing 10% MSC-qualified FBS (Omega Scientific)]. Cultures were maintained in a humidified atmosphere with 5% CO_2_ at 37°C. Approximately 3 weeks after plating, adherent fibroblast-like cells were detached using TrypLE Express for 5 min at 37°C (ThermoFisher) and filtered to remove any tissue fragments. The collected cells were then reseeded and maintained in growth medium containing b-FGF. After 2 weeks of growth with medium replacement every 3 days, the cells were checked for purity by flow cytometry analysis (BD FACSCanto 2 HTS). Cells were assessed for the expression of CD73 (FITC Mouse Anti-Human CD73—BD #561254) and the absence of CD31 (APC-Cy^TM^7 Mouse Anti-Human CD31—BD #563653) and CD45 (APC Mouse Anti-Human CD45—BD #560973). UC-MSC samples displayed CD73 expression in over 90% of cells and lacked expression of CD31 and CD45. Sex of first trimester samples was determined based on PCR for SRY.

### WGS Reanalysis

Whole genome sequencing fastq files from matched, isolated EGFR^+^ and HLA-G^+^ trophoblasts from two patients (11–12 weeks GA) were downloaded from BioProject (accession no. PRJNA445189; Velicky et al., 2018). The fastq files were trimmed (trim_galore v.0.4.1) using a quality score cut-off of 30. The samples were then mapped to GRCh38 using Bowtie2 (v.2.2.7; Langmead and Salzberg, 2012). ERDS 1.1 was used to call CNVs on patient 1 using the erds_pipeline.pl script (Zhu et al., 2012) with default parameters. CNV’s found in both the EGFR^+^ and HLA-G^+^ samples were filtered out. Variants were called using GATK (v 4.0.11.0) on EGFR^+^ and HLA-G^+^ samples from both patients. Briefly, after merging replicate samples, duplicates were marked using Picard (v.2.18.15), base quality scores were recalibrated, and variants were called using HaplotypeCaller. Joint genotyping followed by SNP and InDel recalibration was then performed according to GATK best practices. Low quality variants (GQ < 20.0) were then removed, and resulting vcf files were used to run PURPLE (PURity and PLoidy Estimator; Cameron et al., 2019). To run PURPLE, Amber3 (v.3.1) and Colbalt (v.1.8) were first run in “reference/tumor” mode with the EGFR^+^ sample used as the reference sample and the matched HLA-G^+^ sample used as the “tumor” sample.

### Single-Cell RNA-seq Reanalysis and InferCNV

Single-cell data were downloaded from the European Genome-Phenome Archive (EGA; https://www.ebi.ac.uk/ega/) hosted by the European Bioinformatics Institute (EBI; accession no. EGAS00001002449). Data from only the “normal” placenta samples (PN1, PN2, PN3C, PN3P, and PN4; 38 weeks GA) were used in the InferCNV (inferCNV of the Trinity CTAT Project, https://github.com/broadinstitute/inferCNV) analysis (Patel et al., 2014). PN2 was determined to be an outlier and was removed prior to cell cycle analysis. Prior to running InferCNV, data were analyzed using Scanpy (v.1.4.3; Wolf et al., 2018). Briefly, quality control was performed, and data were filtered and batch corrected before dimensionality reduction and Louvain clustering. Cell cycle analysis was done using Scanpy’s “score_genes_cell_cycle” command and gene sets determined previously (Macosko et al., 2015). Clusters were then annotated by ranking marker genes obtained by performing a modified *t*-test between each cluster and the remaining cells. Sub-clustering was performed on clusters that were not readily identifiable. Two EVT clusters were annotated and used downstream in the inferCNV algorithm as the “tumor” cells. All other annotated cells were considered part of the “normal” reference cells. Anscombe_transform normalization was used before running inferCNV to remove noisy variation at low counts, and the parameter HMM_type “i3” was used to perform inferCNV.

### SNP Genome-Wide Genotyping and CNV Detection

DNA was isolated from placental cell pellets from two first trimester and three term placentae (Qiagen DNeasy Blood and Tissue kit) and quantified (Qubit dsDNA BR Assay Kits, Thermo Fisher Scientific) according to the manufacturer’s protocol. DNA was genotyped using Illumina InfiniumOmni2-5-8v1-4 BeadChips (∼2,381,000 markers with a median spacing of 0.65 kb) at the IGM Genomics Center at UC San Diego. Samples were called in GenomeStudio (Illumina) with an average overall call rate of 99.4%. The CTB sample from one patient (1,391) was removed from the analysis due to a low call rate (91.3%). CNVs were identified using the cnvPartition Plug-in (v.3.2.0) in GenomeStudio. The cnvPartition confidence threshold was set at 100, with a minimum number of SNPs per CNV region of 10. The R (v.3.6.1) package, allele-specific copy number analysis of tumors (ASCAT; v.2.5.2; Van Loo et al., 2010), was used to estimate the ploidy of EVT samples. LogR ratios and BAF values were exported from GenomeStudio, and no GC wave correction was performed. EVT samples were considered “tumor” samples, and matched UC-MSCs were used as “reference” samples.

### Single-Cell CNV Detection

Matched EVTs, CTBs, and UC-MSCs from three term placentae were obtained as detailed above and following isolation were flash frozen. Cells were thawed and immediately resuspended into a single-cell suspension with 1 × PBS and 0.04% BSA and filtered through a Flowmi cell strainer (Belart) before beginning the 10 × Genomics single-cell DNA library prep. Briefly, between 100 and 500 cells in each sample were encapsulated in a hydrogel matrix and lysed, and then the genomic DNA was denatured and captured on a second microfluidic chip in Gel Beads containing unique cell indexes. After creation of amplified barcoded DNA fragments, sequencing libraries were created and sequenced on a NovaSeq at the IGM Genomics Center at UC San Diego. Cell Ranger (v 1.1.0; 10 × Genomics) DNA CNV pipeline was run to associate individual reads back to the individual cell. The reads were mapped to GRCh38, and downstream analysis was performed in LoupeBrowser. Each sample had on average over 1 million mapped and deduplicated reads per cell and a median estimated CNV resolution of less than 1 Mb.

### Flow Cytometric-Based Ploidy Analysis

Matched EVT and CTB and matched EVT, CTB, and UC-MSC were isolated from two first trimester and three term placentae, respectively; three hTSC lines were collected at day 0 and day 5 of EVT differentiation. Cells were washed first with PBS and then cold ethanol. Following the ethanol wash, the cells were allowed to rehydrate in PBS before pelleting and resuspending in 1 ml of a 3 μM DAPI in staining buffer (100 mM Tris, pH 7.4, 150 mM NaCl, 1 mM CaCl_2_, 0.5 mM MgCl_2_, and 0.1% Non-idet P-40) solution. Cells were incubated in the DAPI solution for 15 min at room temperature before filtering and running on a BD FACSCanto II Cell Analyzer.

### FISH

Isolated term placental cell samples from three patients (CTBs, EVTs, and UC-MSCs from each patient) were sent to the Cytogenomics Laboratory at UCSD’s Center for Advanced Laboratory Medicine. Fluorescence *in situ* hybridization (FISH) was performed using enumeration probes for chromosomes 2, 6, 18, and 20 (D2Z2, D6Z1, D18Z1, D20Z1; Abbott Molecular, Inc.). Each probe was examined in 200 interphase nuclei.

### RNA Isolation and RNA-seq Library Construction and Analysis

RNA from 10 first trimester CTB, 10 term CTB, 10 first trimester EVT, and 6 term EVT (split evenly between male and female) were isolated using the mirVana miRNA isolation kit (Ambion). RNA concentration was measured by Qubit RNA BR assay kit (ThermoFisher), and the quality of isolated RNA was checked using a bioanalyzer (Agilent). All samples were found to have a RIN above 7.5. RNA-seq libraries were prepared using the TruSeq Stranded mRNA sample preparation kit (Illumina) at the IGM Genomics Center at UC San Diego. Libraries were pooled and sequenced on NovaSeq 6000 S1Flow Cell (Illumina) to an average depth of 41 million uniquely mapped reads. Quality control was performed using FastQC (v.0.11.8) and multiQC (v.1.6). Reads were mapped to GRCh38.p10 (GENCODE release 26) using STAR (v.2.7.3a; Dobin et al., 2013) and annotated using featureCounts (subread v.1.6.3, GENCODE release 26 primary assembly annotation; Liao et al., 2014). The STAR parameters used were: –runMode alignReads –outSAMmode Full –outSAMattributes Standard –genomeLoad LoadAndKeep –clip3pAdapterSeq AGATCGGAAGAGC –clip3pAdapterMMp 1 – outFilterScoreMinOverLread 0.3 –outFilterMatchNminOverLread 0.3. The featureCounts parameters were: -s 2 -p -t exon -T 13 -g gene_id. Ensembl genes without at least three samples with 5 or more reads were removed from analysis. Normalization and differential expression analysis was performed using the R (v.3.6.3) package DESeq2 (v.1.28.1; Love et al., 2014). Sample sex was accounted for in the DESeq2 design, and, unless otherwise stated, genes with an adjusted *p*-value < 0.05 and log_2_ fold change > 1 were considered differentially expressed. BiomaRt (v.2.42.1) was used to convert Ensembl gene ID’s to HUGO gene names, and Gene Set Enrichment Analysis (GSEA) was done with the R (v.3.6.3) package FGSEA (v.1.14.0) using 10,000 permutations and the Hallmark (v.7.0) pathways gene set, the GO term C5 (v.7.0) gene set, and the transcription factor c3.tft (v.7.2) gene set downloaded from MSigDB. Genes were ranked based on their Wald test statistic after performing differential expression. Additionally, where indicated, founder gene sets for the Hallmark pathway gene sets were downloaded from MSigDB (v.7.2). The cell senescence signature was downloaded from the Human Ageing Genomic Resources^1^ (Tacutu et al., 2018). Cell cycle-related genes for each phase of the cell cycle were previously determined (Macosko et al., 2015). Characterization using principal component analysis (PCA) of the three hTSC lines derived for this study and two previously reported hTSCs (Okae et al., 2018) was done by merging the raw counts from six placental samples (three EVT and three CTB) and four hTSC samples (duplicates of blastocyst derived hTSCs and placental derived hTSCs) from Okae et al., with the nine hTSC samples (triplicates of each hTSC line) and 36 placental samples from this study, filtered as specified above. The combined RNA-seq data were then processed and transformed using DESeq2’s variance stabilizing transformation method before performing PCA. Gene list enrichment analysis was done with Enrichr (Kuleshov et al., 2016). Visualization was performed with the R package ggplot2 or with the python packages seaborn, matplotlib, or plotly.

Gene regulatory networks were created by first performing GSEA using the transcription factor prediction gene set c3.tft (v.7.2) from the Molecular Signatures Database. Transcription factors (TFs) used as input into the gene regulatory network inference algorithm were selected based on adjusted *p*-value (<0.05). Arboreto (v 0.1.5; Moerman, 2019) was run using the GRNBoost2 algorithm. The input into the algorithm consisted of the differentially expressed genes (DEGs, adjusted *p*-value < 0.05 and log_2_ fold change > 1) from a given comparison and the significantly enriched TFs for the same comparison. For each target gene, the algorithm uses a tree-based regression model to predict its expression profile using the expression values of the set of input TFs. The algorithm outputs TF targets with a calculated importance score. The top 1,500 genes by log_2_ fold change were then used to create a protein–protein interaction network using the stringApp [v.1.5.1, confidence (score) cutoff = 0.4, max additional interactors = 0, use smart delimiters] application in Cytoscape (v.3.8.0). The networks were then clustered using MCL clustering with the clusterMaker2 application (v.1.3.1, inflation value = 2.0, assumption that edges were undirected, and loops were adjusted before clustering). The importance scores from the genes in each cluster were then summed to find the TFs with the highest importance for each subcluster.

### RNA Isolation for qPCR of hTSC and EVT Derivatives

RNA was isolated using NucleoSpin^®^ kit (Macherey-Nagel, Duren, Germany), and 300 ng of RNA was reverse transcribed to prepare cDNA using PrimeScript^TM^ RT reagent kit (TAKARA, Mountain Vew, CA, United States) following the manufacturer’s instructions. qRT-PCR was performed using Power SYBR^®^ Green RT-PCR Reagents Kit (Applied Biosystems, Carlsbad, CA, United States) and primers listed in Supplementary Table 2. Data were normalized to beta-actin and shown as fold-change over day 0 (undifferentiated hTSC). Statistical analysis was performed using *t*-test. Data are expressed as mean ± SD of 2^–ddCt^ values. The level of statistical significance was set at *p* < 0.05.

### Western Blot

Human trophoblast stem cell 1,049 cells were differentiated into EVT in 10 cm dishes for 5 days. Cell lysate was collected every day for 5 days using RIPA buffer (Fisher Scientific, United States) containing protease and phosphatase inhibitors (Roche Applied Science, United States) according to the manufacturer’s protocol. Protein concentration was quantified by BCA protein assay (Thermo Scientific, United States). Thirty μg of total protein was loaded onto a 10% denaturing polyacrylamide gel for separation and then transferred to PVDF membranes by electrophoresis. Membranes were blocked with 5% non-fat dried milk in Tris-buffered saline containing 0.1% (v/v) Tween 20 (Sigma-Aldrich) for 1 h and then incubated overnight with primary antibodies: rabbit anti-STAT1 (Cell Signaling Technology or CST #9175) or mouse anti-ACTB (Sigma-Aldrich #A5441). Followed by 1-h incubation with HRP-conjugated secondary antibodies (donkey anti-rabbit IgG, CST #7074S or anti-mouse IgG, CST #7076S), signals were developed using SuperSignal West Dura Extended Duration substrate (Thermo Fisher, United States) and captured on film.

### Immunostaining and *in situ* Hybridization

First trimester placental tissues were fixed in 4% paraformaldehyde in phosphate-buffered saline for 10 min and then permeabilized with 0.5% Triton X-100 for 2 min. Tissues were stained with mouse anti-HLAG antibody (clone 4H84; Abcam) and rabbit anti-STAT1 (CST #9175), using Alexa Fluor-conjugated secondary antibodies (Thermo Fisher), counterstained with DAPI (Invitrogen), and then visualized using a Leica DM IRE2 inverted fluorescence microscope.

Term placenta samples were fixed in neutral-buffered formalin and embedded in paraffin wax. Immunohistochemistry (IHC) and *in situ* hybridization (ISH) were performed on 5-μm sections of these tissues on a Ventana Discovery Ultra automated stainer (Ventana Medical Systems) at the UC San Diego Advanced Tissue Technology Core laboratory. For IHC, standard antigen retrieval was performed for 40 min at 95°C as per the manufacturer’s protocol (Ventana Medical Systems), and the section was stained using mouse anti-HLAG antibody (clone 4H84; Abcam). Staining was visualized using 3,3′-diaminobenzidine (DAB; Ventana Medical Systems), and slides were counterstained with Hematoxylin. For ISH, we used the RNAscope method with probes specific to human GCM1 from ACD-Bio. Following amplification steps, the probes were visualized using DAB, and slides were counterstained with hematoxylin. IHC and ISH slides were analyzed by conventional light microscopy on an Olympus BX43 microscope (Olympus).

## Results

### Term EVT Lacks Recurrent CNVs at Specific Genomic Regions

To address the question whether human EVTs are characterized by specific CNVs or contain whole genome amplifications (polyploidy), we first reanalyzed WGS data from recently published EGFR^+^ (CTB, *n* = 2) and HLA-G^+^ (EVT, *n* = 2) trophoblasts isolated from first trimester placentae (11 and 12 weeks GA; Velicky et al., 2018). To identify CNVs, we applied the Estimation by Read Depth with Single-nucleotide variants (ERDS) algorithm (Zhu et al., 2012), which was recently found to have high sensitivity and accuracy (Trost et al., 2018) and is an orthogonal method to those previously published (Velicky et al., 2018). Our reanalysis found fewer duplications in the HLA-G^+^ samples than in the EGFR^+^ samples; in addition, no duplications encompassed genes previously identified to be contained within amplified genomic regions of murine TGCs (Hannibal and Baker, 2016). Of the 35 genes found to have a duplication unique to the HLA-G^+^ sample, 3 (TTC34, PKP1, and MBD5) were identified in similar previously published data from second trimester whole human placental tissue (Kasak et al., 2015; data not shown). Read depth CNV detection algorithms use intra-chromosomal comparisons and therefore do not provide aneuploidy/polyploidy detection. Therefore, to determine the ploidy of the HLA-G^+^ samples, we applied the PURPLE (PURity and PLoidy Estimator) algorithm to the WGS data (Cameron et al., 2019; Priestley et al., 2019), which reported both HLA-G^+^ samples to be diploid, with no evidence of significant duplications (Supplementary Figure 1A). We do note that the Patient 2 and Patient 1 samples were properly identified as female (two copies of the X chromosome) and male (one copy of the X chromosome), respectively.

**FIGURE 1:**
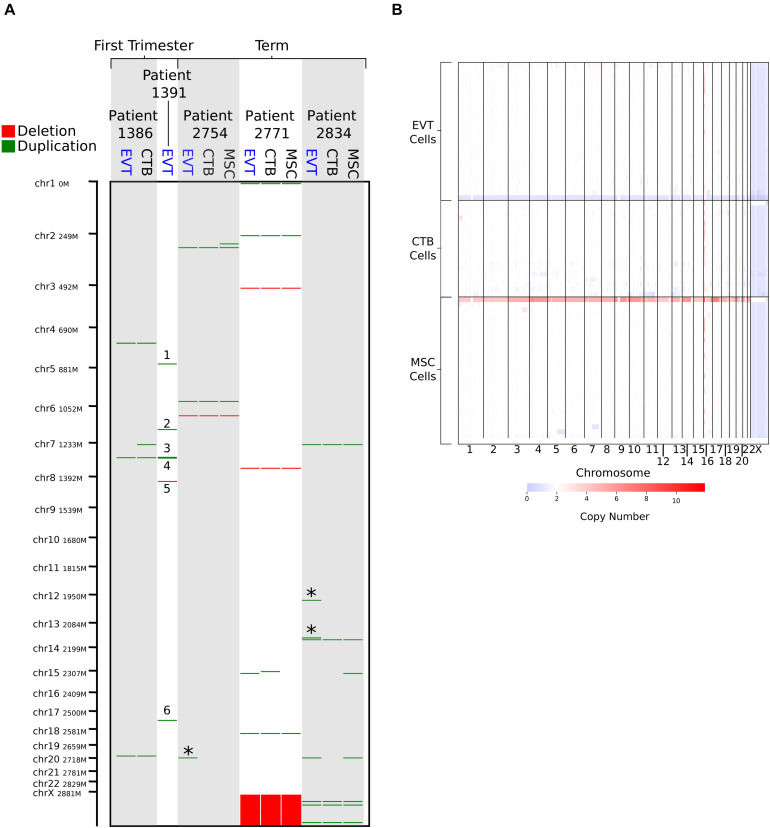
Copy number variation (CNV) analysis using genome-wide SNP genotyping array and single-cell CNV data. **(A)** CNV analysis on matched cytotrophoblast (CTB), extravillous trophoblast (EVT), and umbilical cord-derived mesenchymal stem cells (MSC) from three term placentae, and matched CTB and EVT from two first trimester placentae, using genome-wide SNP genotyping array (CTB sample from Patient 1,391 was removed due to poor data quality). Deletions are shown in red and duplications in green. “^∗^” indicates duplications that are unique to term EVT. Numbered CNVs are those potentially unique to first trimester EVT, though no matched CTB sample was available for Patient 1,391 for direct comparison. Patient 2,771 is female, and all other patients are male. **(B)** CNV calls from single-cell CNV analysis on matched CTB, EVT, and MSC from a single term placenta sample (Patient 2,757—male). Red on the heatmap represents increase in copy number and blue a loss in copy number. White represents a copy number of two.

Next, to find potential CNV genomic hotspots in term EVT, we isolated matched EVTs, CTBs, and UC-MSCs from three term placentae (Supplementary Table 1) and performed CNV calling on data from genome-wide SNP genotyping arrays. For comparison, we performed a similar analysis with matched CTB and EVT from two first trimester placentae. After removing one of the first trimester CTB samples due to inadequate data quality, we found nine CNVs unique to EVT samples (not found in either the matched CTB or the matched UC-MSC samples; Figure 1A). Of these nine CNVs, six were found in the first trimester EVT sample that lacked a matched CTB sample (numbered in Figure 1A), and three were duplications unique to the term EVT samples (green lines marked by ^∗^ in Figure 1A), although not common between term EVT; none of the nine CNVs overlapped with previously identified CNV regions (Kasak et al., 2015). We next sought to determine the ploidy of our EVT samples by running ASCAT (Van Loo et al., 2010) on the data from genome-wide SNP genotyping arrays. We compared the EVT samples to their matched diploid UC-MSCs and again found no evidence of polyploidy in our term EVT samples (Supplementary Figure 1B).

To exclude the presence of a subpopulation of EVT cells showing polyploidy or a significantly higher load of CNVs, we performed single-cell CNV analysis on over 600 matched EVT, CTB, and UC-MSC cells isolated from one term placenta. The estimated ploidy in each of the three samples was the same, at 1.95. Although some cells contained duplications, we were not able to verify the existence of a polyploid subpopulation (Figure 1B). Finally, we interrogated a recently published term placentae single-cell RNA-seq dataset (Tsang et al., 2017) using InferCNV. After identifying EVT cells based on expression of HLAG, we used InferCNV to compare their expression intensity to a set of reference “normal” cells in the same placental samples. We again found no evidence of polyploidy. We did find cells with numerous smaller CNVs in many of the EVT cells, but none that were common across multiple EVT (data not shown). We note that this experiment was performed on one placenta (#2757), and that the placenta was not the same as those used for other analyses.

These data suggest that, while EVT may display CNVs, high frequency CNVs common across EVT within and between different individual placentae might not exist. Moreover, based on reanalysis of existing trophoblast WGS and single-cell RNA-seq data, as well as newly generated bulk SNP genotyping array and single-cell CNV data, we did not observe evidence of polyploidy in term EVT. However, as these techniques are not optimally designed to detect polyploidy, we proceeded with additional analyses to more directly assess this feature.

### Flow Cytometry and Cytogenetic Analysis Confirm the Presence of Polyploid EVT at Term

**FIGURE 2:**
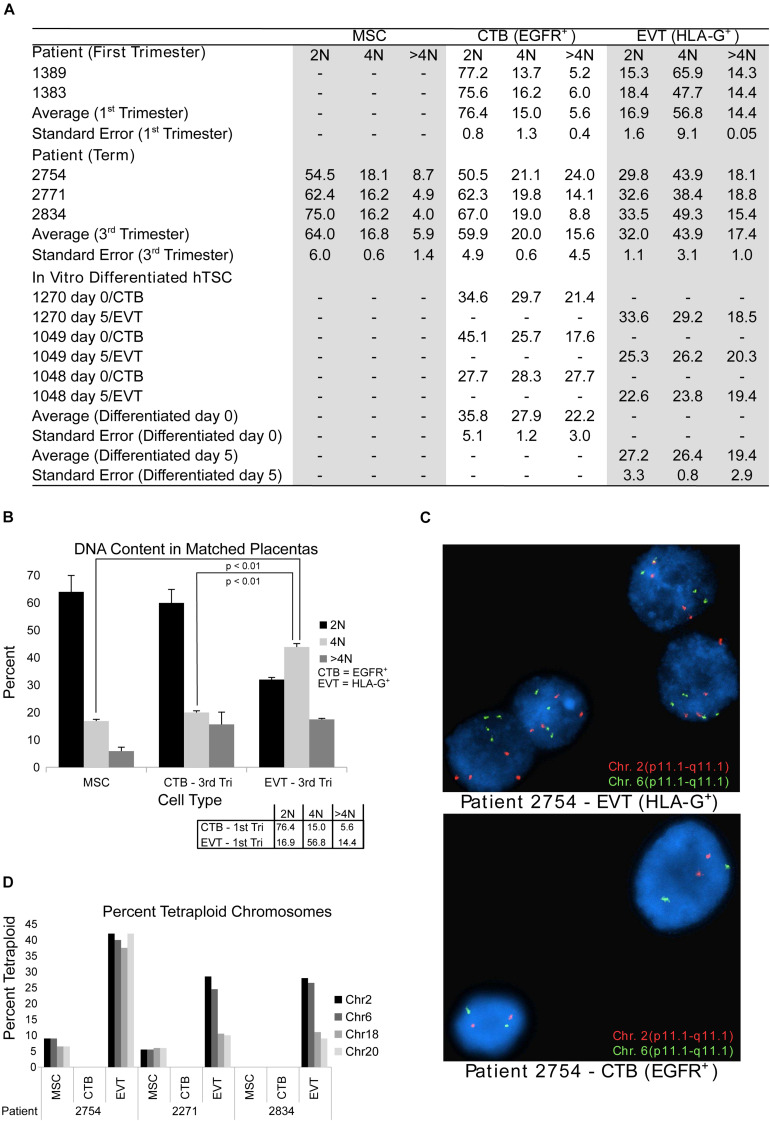
DNA content flow cytometry and cytogenetics analysis. **(A)** Table showing results of DNA content flow cytometry analysis in umbilical cord-derived mesenchymal stem cells (MSC) from term placentae, cytotrophoblast (CTB) and extravillous trophoblast (EVT) from first trimester or term placentae CTB and EVT, and human trophoblast stem cells (hTSCs) that are either undifferentiated (day 0) or differentiated (through a 5-day protocol) into EVT *in vitro*. **(B)** DNA content as determined by flow cytometry from matched CTB, EVT, and MSC from three term placentae. Box in the bottom right corner shows the mean percentage of cells in each ploidy group of matched CTB and EVT from two first trimester placentae as determined by flow cytometry. **(C)** Example images from FISH analysis of matched EVT and CTB from one term placenta. Images show probes targeting chromosome 2 (red) and chromosome 6 (green). **(D)** Bar graph showing the percentage of cells determined by FISH to be tetraploid (chromosomes 2, 6, 18, and 20) in MSC, CTB, and EVT from three term placentae.

Given that the techniques used thus far were performed on bulk cell preparations (and thus might miss genetic alterations present in a subpopulation of component cells), and/or were not designed to detect polyploidy (e.g., single-cell CNV analysis), we next sought to evaluate term EVT using approaches that can reliably detect polyploidy on the single-cell level. First, to confirm the presence of a population of tetraploid first trimester EVT cells, as shown in Velicky et al. (2018), we performed DNA ploidy analysis by flow cytometry on matched CTB and EVT from two first trimester placentae. As reported previously (Zybina et al., 2002; Velicky et al., 2018), the CTBs were predominantly diploid (76% of cells) compared with EVT, of which the majority (57%) were tetraploid (Figure 2A). We also noted a small subpopulation of cells that contained a DNA content above 4N (5.6% in CTB and 14.4% in EVT). We next asked if isolated EVTs from term placentae contain similar proportions of hyperdiploid cells. We found that, although, on average, a lower percentage of term EVT were tetraploid (44%), this was still a significantly larger proportion of tetraploidy than matched CTB and UC-MSC from the same placentae (*p*-value < 0.01; Figures 2A,B). Interestingly, compared with first trimester CTB and term MSC, term CTB showed an almost 3-fold increase in the proportion of >4N cells, and this proportion (∼15%) remained stable in matched term EVT (∼17%; Figures 2A,B). Additionally, we performed *in vitro* differentiation of three hTSC lines into HLA-G^+^ EVT-like cells to assess how closely our *in vitro* differentiated EVT-like cells recapitulated the increased DNA content in primary EVT samples. We found no increase in the percentage of hyperdiploid cells following EVT differentiation of all three hTSC lines; in one cell line (1,049), there was a decrease in the number of diploid cells (and thus an increase in the ratio of polyploid to diploid cells) following differentiation (Figure 2A).

Finally, using the same matched term placental cell isolates, we subjected CTBs, EVTs, and UC-MSCs to FISH analysis using enumeration probes for chromosomes 2, 6, 18, and 20. We again found that our EVT samples had a much higher percentage of tetraploid cells than their matched CTB and UC-MSC samples (Figures 2C,D). We also noted that approximately 7% of EVT cells were called triploid, but no triploid cells were found in any of the CTB or UC-MSC samples (data not shown). Taken together, these data suggest that similar to first trimester EVT, and in contrast to first trimester CTB and third trimester CTB and MSC, term EVTs contain a large subpopulation of polyploid cells. Additionally, the similar proportion of >4N polyploid CTB and EVT at term suggests that this may be a shared feature among term trophoblasts.

### Global Gene Expression Analysis Reveals Unique and Common Pathways Involved in EVT Differentiation and Maturation

**FIGURE 3:**
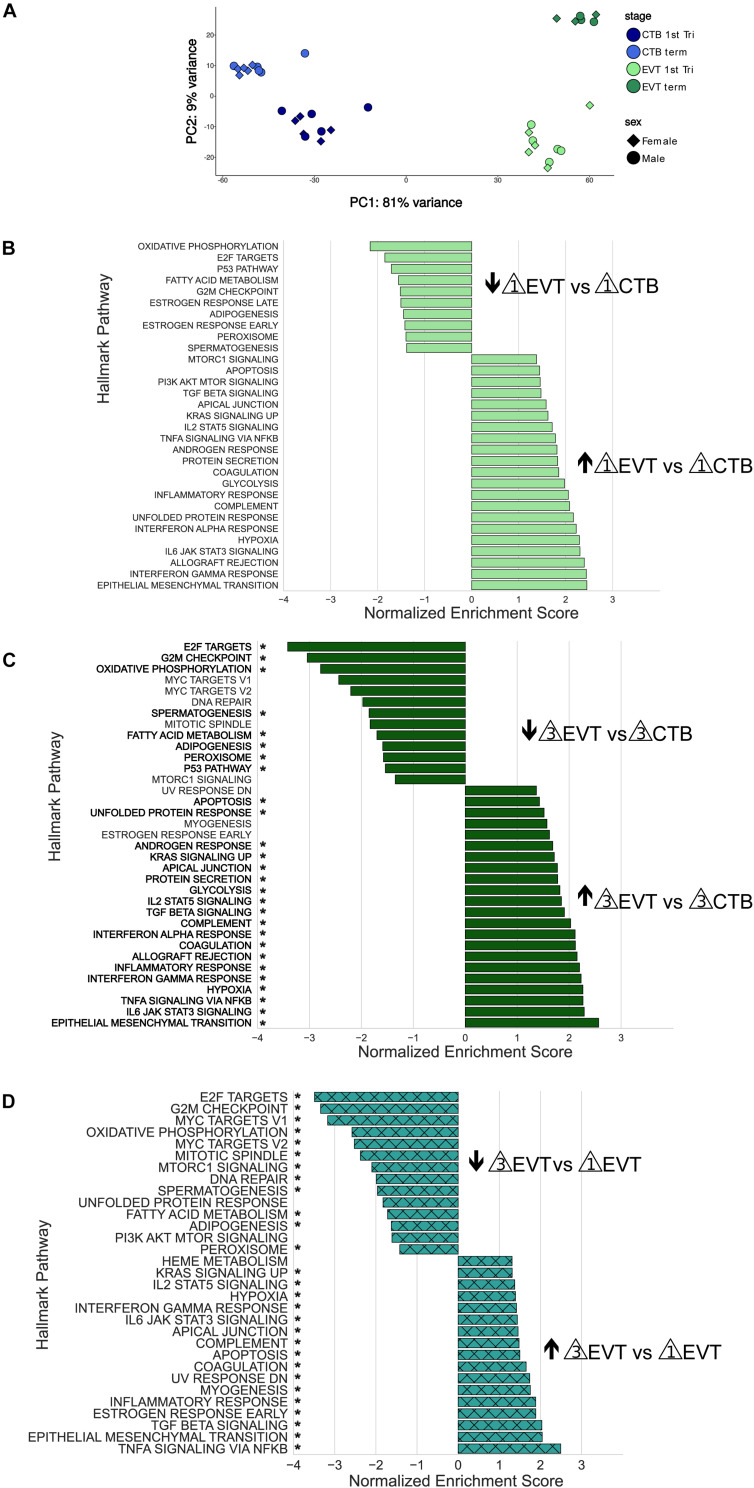
Principal component analysis and Gene Set Enrichment Analysis (GSEA) of RNA-seq data. **(A)** Principal component analysis showing the first two components using all genes post-filtering of all 36 placenta samples (see Supplementary Table 1). Each sample group contained equal numbers of males and females. **(B)** GSEA using the Hallmark pathway gene set of first trimester EVT compared with first trimester CTB. Genes were ranked based on their Wald test statistic after performing differential expression on first trimester EVT and first trimester CTB. Normalized enrichment scores (NES) indicate pathways either up-regulated (NES > 0) or down-regulated (NES < 0) in first trimester EVT vs. first trimester CTB. Only pathways with an adjusted *p*-value < 0.05 are shown. **(C)** GSEA using the Hallmark pathway gene set of term EVT compared with term CTB. Genes were ranked based on their Wald test statistic after performing differential expression on term EVT and term CTB. Normalized enrichment scores (NES) indicate pathways either up-regulated (NES > 0) or down-regulated (NES < 0) in term EVT vs. term CTB. Only pathways with an adjusted *p*-value < 0.05 are shown. Pathway names with an “*” are those that were also found to be significantly enriched in first trimester EVT vs. first trimester CTB. **(D)** GSEA using the Hallmark pathway gene set of term EVT compared with first trimester EVT. Genes were ranked based on their Wald test statistic after performing differential expression on term EVT and first trimester EVT. Normalized enrichment scores (NES) indicate pathways either up-regulated (NES > 0) or down-regulated (NES < 0) in term EVT vs. first trimester EVT. Only pathways with an adjusted *p*-value < 0.05 are shown. Pathway names with an “*” are those that were also found to be significantly enriched in term EVT vs. term CTB.

To further probe the differences between diploid CTB and polyploid EVT, we profiled the transcriptomes of both first trimester and term CTB and EVT. We isolated CTB and EVT from 10 first trimester placentae, CTB from 10 term placentae, and EVT from 6 term placentae (Supplementary Table 1), with equal numbers of male and female placentae, and performed RNA-seq. PCA showed that samples clustered into the four expected groups (first trimester CTB, term CTB, first trimester EVT, and term EVT), based on cell-type along the first principal component, and on GA along the second principal component (Figure 3A). There did not appear to be any obvious transcriptional differences based on sex. However, because we had sequenced a sufficient number of patient samples and had equal numbers of male and female placentae, we performed differential gene expression analysis between male and female EVT (adjusted *p*-value < 0.05, Log_2_ fold change > 1, and normalized mean expression in group > 100) from both GAs. We found a small number of sex-specific DEGs in first trimester EVT, with just 5 genes up-regulated in female EVT, including XIST and EPPK1, the latter a negative regulator of epithelial cell migration, and 11 genes up-regulated in male EVT, all of which were located on the Y chromosome except for PLXDC2 (Supplementary Figure 2A). Interestingly, term EVT showed a larger number of sex-specific DEGs, with 27 genes up-regulated in the female samples and 24 in the male samples (Supplementary Figure 2B). Several of the genes on the Y chromosome were up-regulated in both male term EVT and male first trimester EVT, but no significant gene ontology enrichment was identified in any groups.

To identify differences among these four groups of cells (first trimester CTB and EVT and term CTB and EVT), we first performed differential expression analysis, ranked genes based on their Wald test statistic, and conducted GSEA. CTBs are the proliferative epithelial cells of the placenta and differentiate early in pregnancy into EVT; therefore, we first sought to identify pathways that were significantly enriched between first trimester CTB and EVT (Figure 3B). Similar to previously published microarray data (Apps et al., 2011;Telugu et al., 2013;Tilburgs et al., 2015), all seven of the pathways that make up the immune process category in the Hallmark gene set (Liberzon et al., 2015) were significantly up-regulated in EVT vs. CTB in the first trimester (adjusted *p*-value < 0.05; Figure 3B). Furthermore, similar to what we previously found using gene expression microarrays (Wakeland et al., 2017), pathways such as hypoxia, UPR, and mTOR signaling were significantly up-regulated, and pathways such as oxidative phosphorylation, P53, fatty acid metabolism, and those related to cell cycle control were significantly down-regulated, in first trimester EVT (Figure 3B).

We next analyzed the pathways that were significantly enriched in term EVT compared with term CTB (Figure 3C). Perhaps unexpectedly, nearly all the pathways up-regulated in term EVT were also significantly up-regulated in first trimester EVT (Figure 3C, highlighted by ^∗^). Likewise, most pathways down-regulated in term EVT were also similarly altered in the first trimester comparison (Figure 3C, highlighted by ^∗^). However, in addition to the E2F targets, G2M checkpoint, and P53 pathways, term EVT also showed down-regulation of the three remaining pathways in the proliferation process category (namely: MYC targets V1, MYC targets V2, and mitotic spindle) in the Hallmark gene set (Liberzon et al., 2015).

Next, we examined the EVT maturation process by comparing gene expression between first trimester and term EVT samples (Figure 3D). We again saw many of the same pathways enriched in term EVT, compared with first trimester EVT, as in the comparison with term CTB (Figure 3D, highlighted by ^∗^). We noted that compared with first trimester EVT, term EVT down-regulated all of the proliferation process category pathways (E2F targets, G2M checkpoint, MYC targets, and mitotic spindle), except the P53 pathway gene set (Figure 3D).

**FIGURE 4:**
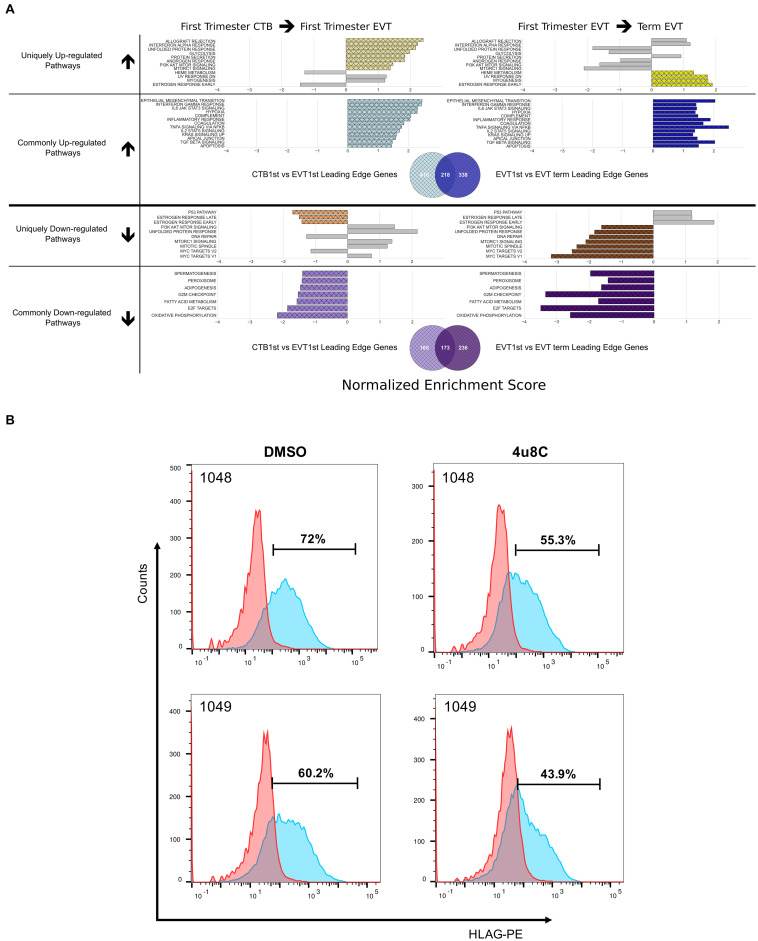
Pathways enriched in the EVT differentiation and maturation process. **(A)** Pathways enriched either in one (unique) or both (common) steps of EVT differentiation (first trimester CTB → first trimester EVT) and maturation (first trimester EVT → term EVT). Colored bars in grid show significantly enriched pathways (adjusted *p*-value < 0.05) that are either uniquely up- or down-regulated in first trimester EVT when compared with first trimester CTB (left side) or uniquely up- or down-regulated in term EVT when compared with first trimester EVT (right side). Likewise, common up- and down-regulated pathways are shown in both comparisons. The gray bars on each unique pathway bar graphs represent the enrichment score of the comparison on the opposite side of the graph to highlight the similarity or difference of the corresponding comparison. The gray bars may show enrichment in the same direction as the colored bars; however, the pathway is considered uniquely regulated because the gray bars represent enrichment that did not achieve statistical significance. The Venn diagrams below each set of bar graphs show the number of unique and shared leading-edge genes in the commonly up-regulated or commonly down-regulated Hallmark pathways. **(B)** Validation of the IRE1-alpha arm of the unfolded protein response (UPR) pathway regulating surface HLA-G expression in EVT. Two hTSC lines were differentiated to EVT *in vitro* in the presence of either the IRE1-alpha inhibitor, 4u8c, or DMSO carrier alone. Graph shows the percentage of HLA-G^+^ cells at the end of the 5-day treatment, with ∼25% decrease in these cells in the presence of the inhibitor.

**FIGURE 5:**
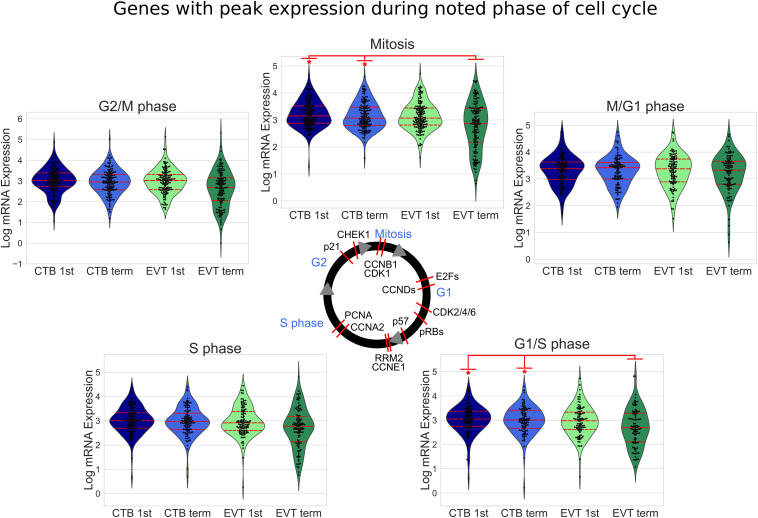
Cell cycle-related gene expression in first trimester and term CTB and EVT. Expression of known cell cycle phase-related genes (Macosko et al., 2015) is shown. The median value of each gene in each group was used to create swarm/violin plots. Inner red dotted lines in swarm/violin plots represent the quartiles of the distribution (*p*-value: * <0.05 by *t*-test). Red lines on cell cycle figure represent location during cell cycle with reported peak expression of the labeled gene (protein names have been used in certain cases to represent multiple genes or because they are more commonly used).

To better understand how specific pathways were regulated during EVT development, we next looked at the pathways that were unique or common through both steps of the EVT maturation process (first trimester CTB → first trimester EVT → term EVT; Figure 4A). Of the common down-regulated pathways in EVT, the cell proliferation pathways E2F targets and G2M checkpoint had the lowest scores. We therefore repeated GSEA using just the founder gene sets (Liberzon et al., 2015) for these two pathways. We found that in both comparisons, term EVT showed significant downregulation for the neighborhood of CCNA2 (Cyclin A2), PCNA, and RRM2 in the GNF2 expression compendium (Supplementary Figure 3A and Figure 5; all comparisons with NES > 3.34 and adj. *p*-value < 0.006). These three genes are expressed just before the onset or during the S phase of the cell cycle, consistent with the absence of cells in the S phase in term EVT.

Two of the Hallmark pathways that were found to be uniquely up-regulated only in the first trimester EVT (compared with term EVT) were the PI3K/AKT/mTOR signaling and UPR pathways (Figures 3B, 4A). These two pathways appeared to switch directions during this two-step process: first up-regulated during the initial conversion of first trimester CTB to EVT and then down-regulated in the subsequent maturation step from first trimester EVT to term EVT. The PI3K/AKT/mTOR pathway has been shown to be involved in the initial transition of CTB to EVT reviewed in Pollheimer and Knofler (2005) and Ferretti et al. (2007), specifically by promoting EMT. The other significantly up-regulated pathway, UPR (Supplementary Figure 3B), is mediated by endoplasmic reticulum (ER) stress and is a method used by cells to detect, eliminate, and avoid further accumulation of misfolded proteins in the ER lumen, which build up due to several environmental cues, including hypoxia, a known EVT differentiation cue (Wakeland et al., 2017). Moreover, UPR is a stress response phenotype, triggered by similar inducers to senescence, and recently found to be present in all types of senescence (Pluquet et al., 2015). We have previously identified this pathway among those up-regulated during the transition from CTB to proximal column EVT (Wakeland et al., 2017); however, it has not been further validated in EVT differentiation and/or function. We therefore repeated GSEA using just the founder gene sets (Liberzon et al., 2015) for the UPR pathway and found that the Reactome activation of chaperone genes by XBP1S (adj. *p*-value < 0.02) was significantly enriched. Furthermore, we found that the GO term IRE1 mediated UPR to be significantly up-regulated in first trimester EVT compared with first trimester CTB (adj. *p*-value < 0.04). Therefore, to assess the importance of the UPR pathway in EVT, we asked whether suppression of the IRE1-alpha arm, which is responsible for activating XBP1(S), would affect EVT formation *in vitro*. We derived two separate hTSC lines from early first trimester placentas; these lines appeared transcriptionally very similar to hTSC lines previously derived from early gestation placentas (Okae et al., 2018; Supplementary Figure 4). We then applied the IRE1-alpha arm inhibitor 4u8c to both hTSC lines during differentiation into EVT and found an ∼25% decrease in the percentage of HLA-G^+^ cells at the end of the protocol in two separate hTSC lines (Figure 4B). However, qPCR did not show alteration of expression of any other EVT marker with 4u8C suppression (Supplementary Figures 5A,B). In addition, only total (and not spliced) XBP1 was increased with EVT differentiation (Supplementary Figure 5C). These results suggest that at least the IRE1-alpha arm of the UPR pathway is needed for proper surface expression of HLA-G but is not required for EVT differentiation *per se*. Overall, these data provide, for the first time, a global look at pathways involved in EVT differentiation and maturation, identifying pathways that are uniquely and commonly up- or down-regulated in either step.

### Transcriptome Analysis Suggests Cell Cycle Arrest, Cellular Senescence, and Endoreduplication as Key Features of EVT

**FIGURE 6:**
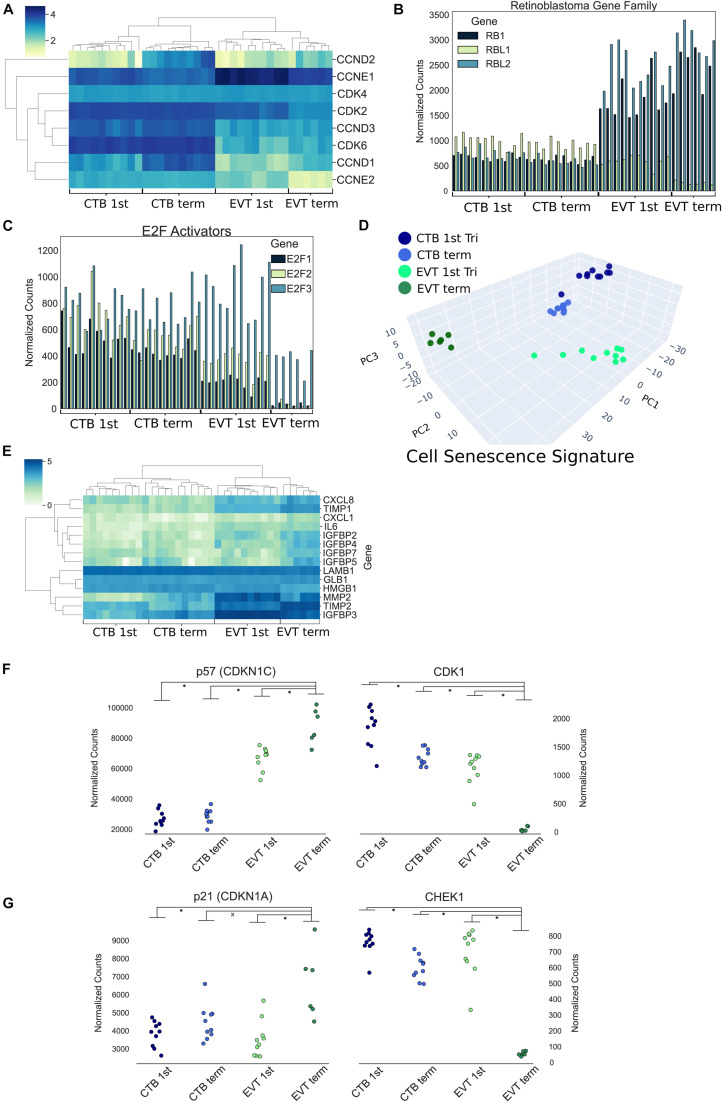
Cell cycle and endoreduplication gene expression. **(A)** Heatmap of G1 phase cyclins and cyclin-dependent kinases using log transformed normalized gene counts. **(B)** Bar graph showing retinoblastoma family genes (*RB1*, *RBL1*, and *RBL2*) normalized gene expression. **(C)** Bar graph displaying E2F activators (E2F1, E2F2, and E2F3) normalized gene expression. **(D)** Three-dimensional principal component analysis using genes (*n* = 1,225) associated with human cellular senescence signature. **(E)** Heatmap of previously identified (Basisty et al., 2020) senescence-associated secretory phenotype (SASP)-associated genes using log transformed normalized gene counts. **(F)** Normalized expression of p57 (CDKN1C) and CDK1 (*p*-value: ^∗^<0.01 by *t*-test). **(G)** Normalized expression of p21 (CDKN1A) and CHEK1 (*p*-value: ^∗^<0.05 by *t*-test).

The induction of cellular senescence leads to irreversible growth arrest and has been proposed as a ploidy-limiting mechanism (Hayflick, 1965; Johmura and Nakanishi, 2016). Our initial transcriptomic analysis using GSEA (Figures 3B–D), along with the evaluation of expression of mitosis and cellular proliferation-associated genes (Figure 5), suggested that term EVTs are not cycling. We found that genes associated with all phases of the cell cycle exhibited decreased expression in term EVT, with the largest difference seen in genes associated with mitosis (Figure 5). An active cell cycle is characterized by expression of cyclins and cyclin-dependent kinases (CDKs). G1 phase cyclins and CDKs were differentially expressed between EVT and CTB samples, with the lowest overall expression in term EVT, suggesting G1 cell cycle arrest (Figure 6A). Additionally, two of the three retinoblastoma family genes, *RB1* and *RBL2*, both shown to play pivotal roles in the negative control of the cell cycle by binding to E2F TFs and thus preventing S phase entry (Giacinti and Giordano, 2006), were significantly up-regulated (adj. *p*-value < 0.01) in EVT compared with CTB (Figure 6B). Additionally, the genes encoding the “activating” E2Fs, which are known to interact with RB proteins to restrict cell cycle advancement (Shay et al., 1991) were significantly lower in term EVT than in CTB (adj. *p*-value < 0.01; Figure 6C). Interestingly, mitosis-associated cyclin B (CCNB1) was highly expressed in first trimester EVT (Velicky et al., 2018), but was roughly 100-fold lower in term EVT, suggesting the absence of mitosis in term EVT (Supplementary Figure 6A). Moreover, expression of the mitosis-linked genes, *CDK1*, *MKI67*, and *AURKA* (Zybina et al., 2004), was >10-fold lower in term EVT than in the other three groups (Supplementary Figure 6A). Additionally, we performed cell cycle scoring on term single-cell RNA-seq data (Tsang et al., 2017) and found that the EGFR^+^ cluster contained a much higher fraction of cells in the G1 and S phases compared with the HLA-G^+^ cluster, which was contained predominantly in the G2/M phase, as previously reported (Velicky et al., 2018; Supplementary Figure 6B). Next, to investigate whether polyploid EVT displayed a senescence-like transcriptomic profile, we performed PCA using genes (*n* = 1,225) reported to comprise a human senescence transcriptomic signature (Tacutu et al., 2018). We found that term EVT samples were uniquely clustered away from the other cell types, with first trimester EVT samples closer to both CTB groups than to the term EVT group (Figure 6D). A similar clustering was not present in a PCA plot using a random set of 1,225 genes (data not shown). Furthermore, the gene (*GLB1*) encoding the senescence-associated marker Beta-Galactosidase (SAβG; adj. *p*-value < 0.001), along with several other senescence-associated secretory phenotype (SASP) and metabolic genes (Basisty et al., 2020), was most highly expressed in either first trimester or term EVT (Figure 6E). Taken together, these results suggest that EVTs are undergoing cell cycle arrest and senescence.

A recent study has suggested that first trimester EVTs induce endocycles and enter a senescent state (Velicky et al., 2018). Endoreduplication consists of DNA replication without cell or nuclear division. It is thought to be triggered by inhibition of CDK1 by p57 (CDKN1C) and suppression of checkpoint protein kinase (CHEK1) by p21 (CDKN1A), preventing induction of apoptosis (Ullah et al., 2008). In our data, we noted the strongest reciprocal expression of p57 and CDK1, as well as p21 and CHEK1, in term EVT (*p*-value < 0.05; Figures 6F,G). Endoreduplication is also characterized by downregulation of CDK1, Cyclin A, and Cyclin B, with simultaneous persistence of Cyclin E expression (Ullah et al., 2009). In our data, CDK1 was uniquely decreased in term EVT (Figure 6F); however, both Cyclin B (CCNB1) and Cyclin E1 (CCNE1) were highly expressed in first trimester EVT, with CCNB1 expression plummeting and CCNE1 persisting, albeit at a lower level, in term EVT (Supplementary Figures 6A,C). Although Cyclin A (CCNA1) had a similar expression profile to Cyclin E1, it was expressed at an extremely low level throughout (data not shown). This pattern of gene expression is most consistent with endoreduplication occurring in some first trimester EVT but becoming more ubiquitous/pronounced in term EVT.

### TF Drivers Characteristic of EVT

**FIGURE 7:**
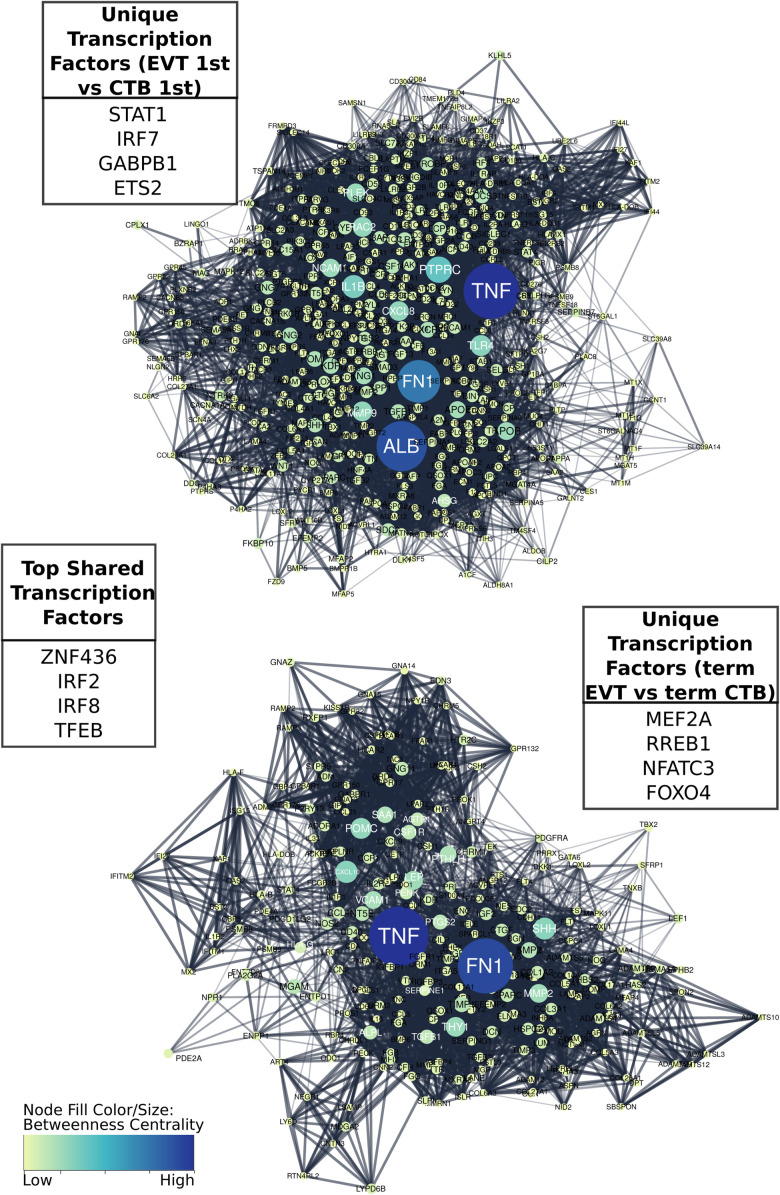
Comparison of gene regulatory networks involved in development of first trimester and term EVT. Networks were created by first generating protein to protein interaction STRING networks of up-regulated genes, either in first trimester EVT compared with first trimester CTB (top), or in term EVT compared with term CTB (bottom), then clustering networks into subnetworks. The largest subnetwork from each comparative analysis is shown. Displayed “unique” transcription factors (TFs; top left and bottom right tables) were those found to have the highest summed importance scores in terms of the labeled subnetwork. Displayed “top shared” TFs (middle table) were the top four transcription factors in terms of importance for both subnetworks. Subnetwork node sizes and colors are determined by the nodes calculated betweenness centrality scores.

To better understand the TF regulatory drivers of first trimester and term EVT, we performed GSEA using the TF prediction gene sets from the Molecular Signatures Database. To determine which enriched TFs were critical in each set of DEGs, and to infer gene regulatory networks, we used a tree-based regression model to calculate an “importance score” for each TF gene target pair using GRNBoost2 in the Arboreto software library (Moerman, 2019). Next, we created STRING networks for each of the top 1,500 DEGs in each of the different cell type comparisons and clustered the networks into subnetworks. We were then able to use the TF gene target importance scores to infer which TFs were critical to each of the subnetworks. In this process, more than one subnetwork may be assigned to a given TF. We first asked which TFs had the highest importance scores when assessing the differentiation of CTB to EVT, initially focusing on the paired first trimester cells and evaluating genes up-regulated in EVT over CTB. Following network clustering, these genes clustered into several subnetworks, with the largest containing close to 1,000 genes with *TNF*, *FN1*, and *ALB* as the genes with the highest centrality scores (Figure 7, top), and the top four TFs being *STAT1*, *IRF7*, *GABPB1*, and *ETS2* (Figure 7, top).

Next, we evaluated the TF network up-regulated in EVT compared with CTB at term. Following clustering, we compared the largest subnetwork in this comparison to the largest subnetwork up-regulated in first trimester EVT (compared with first trimester CTB) and found that approximately 25% of the genes were the same. The top four TFs unique to the largest term EVT subnetwork were MEF2A, RREB1, NFATC3, and FOXO4 (Figure 7, bottom). Additionally, *FN1*, the highest expressed gene in our EVT samples, and *TNF* appeared to again have the highest centrality scores in this subnetwork (Figure 7, bottom). The top ranked TFs in terms of importance scores shared in these two largest subnetworks in both first trimester and term EVT were ZNF436, IRF2, IRF8, and TFEB (Figure 7, center).

**FIGURE 8:**
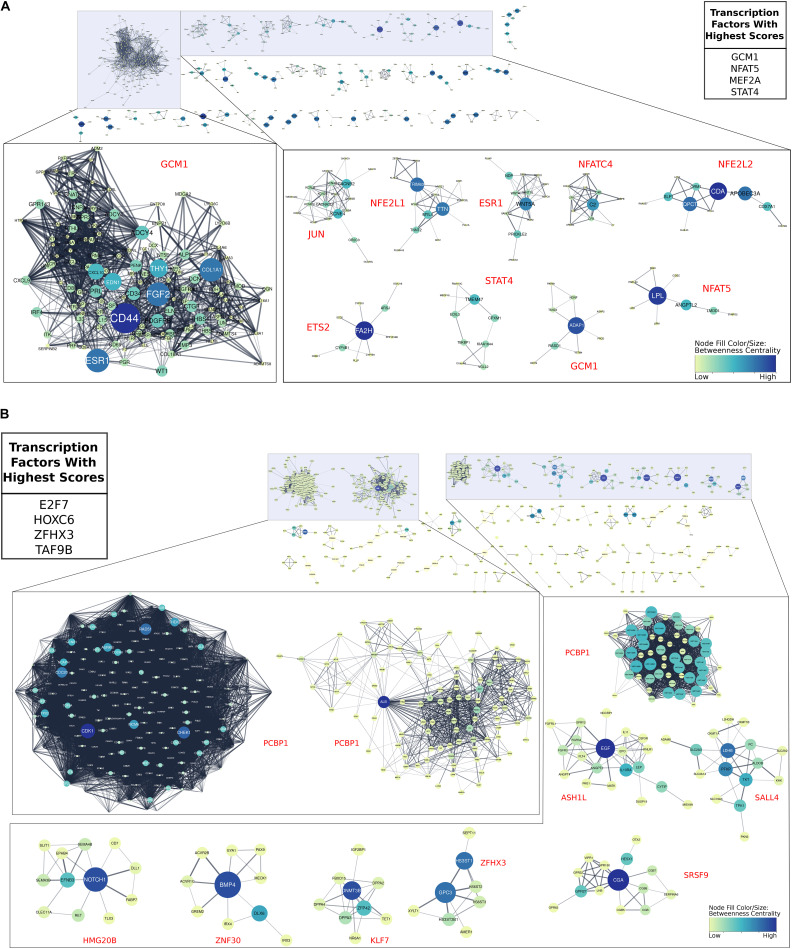
Gene regulatory networks involved in EVT maturation. All gene regulatory subnetworks were created by clustering STRING networks of up- and down-regulated genes in term EVT compared with first trimester EVT. **(A)** All subnetworks created from genes up-regulated in term EVT compared with first trimester EVT. The top 10 largest subclusters are enlarged. The top four transcription factors (TFs) with the highest summed scores for all genes in the comparison are shown in the top right table. **(B)** All subnetworks created from down-regulated genes in term EVT compared with first trimester EVT. The top 10 largest subclusters are enlarged. The top four TFs with the highest summed scores for all genes in the comparison are shown in the top left table. For both analyses, the red TF names alongside each subnetwork are those with the highest importance scores in that subnetwork. Subnetwork node sizes and colors are determined by the nodes calculated betweenness centrality scores.

We next asked which TFs were important for EVT maturation. Using the genes that were up-regulated in term EVT, compared with first trimester EVT, we found that the four TFs with the highest importance scores were GCM1, NFAT5, MEF2A, and STAT4 (Figure 8A). GCM1 had decreased expression in term EVT compared with first trimester EVT but was also the TF with the highest importance score in the largest subnetwork (Figure 8A). This subnetwork was enriched for genes in the PI3K/AKT/mTOR pathway and extracellular matrix organization (adj. *p*-value < 0.01). We then analyzed which TFs had the highest importance scores when comparing genes down-regulated in term EVT, compared with first trimester EVT. The top four TFs were E2F7, HOXC6, ZFHX3, and TAF9B (Figure 8B). Additionally, we found that PCBP1 was the TF with the highest importance score in the three largest subnetworks in this comparison (Figure 8B). The identification of TFs involved in EVT differentiation and maturation provides the first step toward the ability to model this important placental cell type *in vitro* and to begin to decipher the various functions these cells serve at the maternal–fetal interface.

**FIGURE 9:**
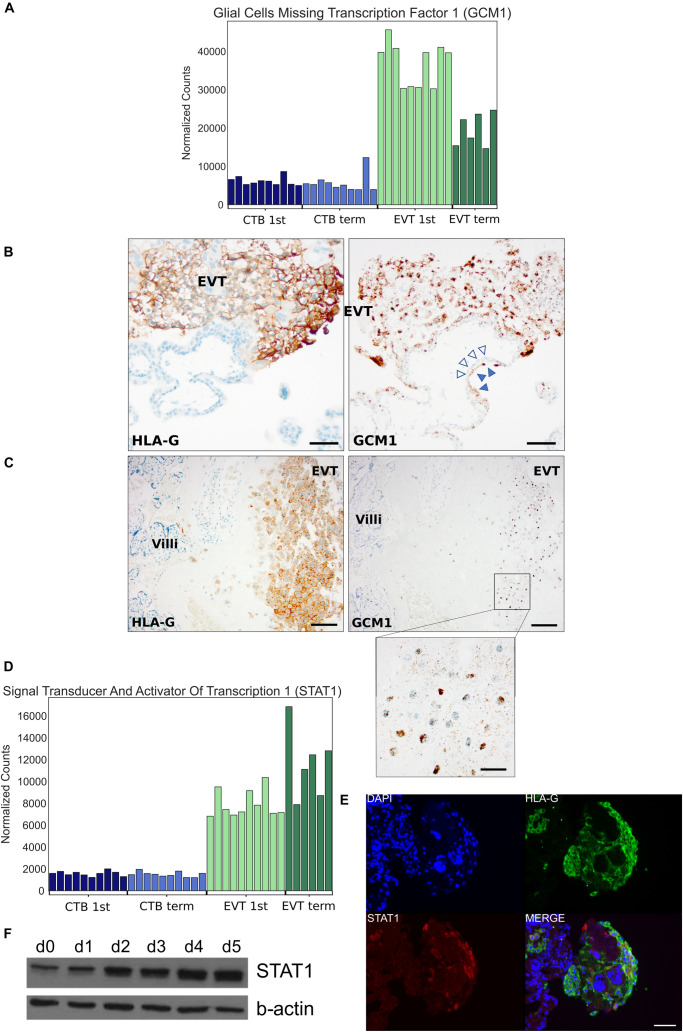
Localization and expression of GCM1 and STAT1 transcription factors. **(A)** Bar graph depicting GCM1 expression in all 36 placental samples using RNA-seq. **(B)** *In situ* hybridization of GCM1 (right-side) and immunohistochemistry for HLA-G (left-side) in adjacent sections of first trimester EVT. Empty arrowheads point to CTB and filled arrowheads to syncytiotrophoblast, which also expresses GCM1. HLA-G staining highlights EVT. Scale bars = 50 μm. **(C)** *In situ* hybridization of GCM1 (right-side, with further magnification in inset) and immunohistochemistry for HLA-G (left-side) in adjacent sections of term EVT. HLA-G again highlights EVT at the basal plate. Scale bar = 100 μm in main panels and 25 μm in inset. **(D)** Bar graph depicting STAT1 expression in all 36 placental samples using RNA-seq. **(E)** Immunostaining of first trimester placenta with antibodies against HLA-G and STAT1 and counterstained with DAPI. Scale bars = 50 μm. **(F)** Western blot of STAT1 and beta-actin (control) during EVT differentiation of 1049 hTSC line.

To validate some of these findings, we chose to focus on two TFs: STAT1, because it was identified in the top four TFs of the largest TF subnetwork in first trimester EVT (Figure 7, top), and GCM1, because it was within the top four TFs with the highest importance scores in term, compared with first trimester EVT, and the TF with the highest importance score in the largest subnetwork (Figure 8A). We first evaluated GCM1 expression and confirmed that this gene is most enriched in first trimester EVT with levels decreased at term (Figure 9A). We performed ISH on first trimester and term placental sections and confirmed enrichment of this gene to be highest in first trimester HLA-G^+^ EVT (Figures 9B,C). We next confirmed STAT1 gene expression in first trimester and term CTB and EVT and found that in fact it is enriched in EVT, with similar levels at the different gestational timepoints (Figure 9D). We stained first trimester placental tissues with antibodies to HLA-G and STAT1 and found that STAT1 expression was confined to HLA-G^+^ cells (Figure 9E). We also differentiated one of our primary hTSC lines into EVT and found that STAT1 expression significantly increases over this differentiation time course (Figure 9F). These data confirm that our analyses have indeed identified novel TF drivers of EVT differentiation and/or function. Future studies are needed to further validate the numerous additional findings from our TF network analyses, and to functionally assess the role of each of these TF’s in EVT.

## Discussion

Abnormal placental development has been linked to numerous pregnancy complications, including pre-eclampsia, intrauterine growth restriction, miscarriage, and stillbirth (Khong, 1986; Khong et al., 1987; Hustin et al., 1990; Brosens et al., 2002; Fisher, 2015; Knofler et al., 2019). The placenta develops by forming primary villi consisting of rapidly proliferating CTB. These cells fuse to form an outer layer of villous syncytiotrophoblast. At the same time, the CTBs start to differentiate into EVT within the trophoblast columns of the early gestation placenta, anchoring the placenta to the uterine wall (Turco and Moffett, 2019). EVTs mature as they move distally within the trophoblast column, and subsequently invade into the decidua and myometrium as interstitial EVT, or remodel decidual arterioles as endovascular EVT (Pijnenborg et al., 1980, 2006). While much has been done to characterize early gestation EVT, fewer studies have focused on mature EVT. In this study, we set out to characterize these cells from normal term placentae, including their genome and transcriptome; given this tissue source, the majority of these cells are likely mature interstitial (and not endovascular) EVTs. Additionally, by comparison to both first trimester and term CTB, as well as first trimester EVT, we assembled gene regulatory networks to better understand the pathways and TFs involved in the maturation and unique genomic architecture of EVT.

### Characterization of EVT Genome

Extravillous trophoblasts share numerous cellular characteristics with tumor cells, including EMT (Viovac and Aplin, 1996). In addition, multiple studies have suggested that EVTs are tumor-like in their carriage of genomic aberrations (Zybina et al., 2002, 2004; Weier et al., 2005; Velicky et al., 2018). Specifically, earlier studies (Zybina et al., 2002, 2004) suggested that EVTs show moderate genome amplification (up to 8N) but are not highly polyploid, unlike mouse TGCs; the latter not only show significant polyploidy (with some cells > 900N) but also contain functionally relevant under- or over-represented genomic regions (Hannibal et al., 2014; Hannibal and Baker, 2016). Another study suggested the presence of aneuploidy and hyperdiploidy in trophoblast, with the greatest proportion in HLA-G^+^ EVT (Weier et al., 2005). Most recently, Velicky et al. (2018) have reported that the majority of first trimester EVTs were hyperdiploid, possibly through endoreduplication, and undergoing senescence through this process. Several recent studies have also examined the existence of CNVs in the placenta (Kasak et al., 2015; Meinhardt et al., 2015; Coorens et al., 2021), but with inconsistent conclusions. One study identified amplification of the *ERBB2* gene and found it to be particularly prominent in EVT (Meinhardt et al., 2015). Other studies have used bulk placental samples with parental controls and identified widespread copy number changes; however, these studies either excluded EVT from samples or could not confirm the presence of somatic genomic rearrangements in placenta-specific cell types at term (Kasak et al., 2015; Coorens et al., 2021).

Here, to gain a better understanding of the genomes of normal human EVT, we applied multiple cellular and bioinformatic methods. First, we reanalyzed a previously published WGS dataset from CTB and EVT purified from first trimester placentae (Velicky et al., 2018), applying CNV detection algorithms not used in the original analysis. We did not find CNVs that had been reported in mouse TGCs but did find three duplications previously reported in bulk second trimester human placental samples (Kasak et al., 2015; Coorens et al., 2021). Additionally, we performed genome-wide CNV analysis of our own samples using high-resolution SNP genotyping arrays and identified three duplications in our term EVT samples, none of which were common between samples or previously reported (Kasak et al., 2015; Coorens et al., 2021). Although we could not identify any common EVT-specific CNVs among preparations from different placentae, including those from a previous publication, definitive assessment of this observation will require a substantially larger sample size. We also applied ploidy-detection algorithms on Velicky et al. (2018)’s WGS data as well as our SNP genotyping data but found no evidence of polyploidy. Lastly, to rule out the presence of a subpopulation of EVT cells showing a high polyploidy rate or widespread CNVs, we performed single-cell CNV analysis on a set of isolated samples and again found no evidence of polyploidy or cells with a large number of CNVs. Nevertheless, we suspect that the algorithms used in our analysis are poorly suited to calling polyploidy.

To validate previous reports of polyploidy in EVT, and to confirm the limitations of the algorithms applied to our genomic data, we determined the DNA content of our isolated placental cells by flow cytometry and found the majority (57%) of first trimester EVT and a lower percentage (44%) of term EVT to be tetraploid. FISH confirmed our flow cytometry results. As previously reported (Zybina et al., 2002), we also noted a population of EVT with > 4N status, with a slightly larger proportion of such cells at term. Interestingly, a similar proportion of term CTB (∼15%) had a >4N status, suggesting that this may be a feature of “aged” trophoblast, as recently reported (Coorens et al., 2021), rather than a unique feature of term EVT. Additionally, we differentiated primary hTSC cells to EVT, using established protocols (Okae et al., 2018) to assess how well current *in vitro* models recapitulate the polyploid phenotype seen in primary EVT. We found that *in vitro* differentiation did not increase the proportion of hyperdiploid cells, suggesting that perhaps *in vivo*, EVTs receive additional signals from their environment that lead to polyploidization. Additional work is thus needed to better recapitulate the *in vivo* EVT state *in vitro*.

The biological significance of polyploidization remains unclear, particularly in the placenta. In the liver, polyploidization has been hypothesized to be a hallmark of terminal differentiation, a mechanism through which a cell may shift energy usage from cell division to more important functions, and/or as a way to protect cells against oxidative stress and genotoxic damage (Wang et al., 2017). Oxygen tension in blood surrounding the placental villi has been reported to increase threefold during pregnancy causing oxidative stress (Jauniaux et al., 2000) and recently (Coorens et al., 2021) reported a substantial mutational burden in placental tissue. Thus, the onset of endoreduplication and senescence, which requires replication arrest in a previously proliferative cell type such as EVT, would lead to the acquisition of multiple sets of chromosomes and could function to buffer cells against harmful mutations. Our evaluation of EVT transcriptome, discussed below, may shed some light on this question; however, future studies examining transcriptomes of EVT subpopulations, separated based on different levels of ploidy, along with delineation of the spatial distribution of these subpopulations, are needed to more precisely define the function(s) of polyploid EVT.

### Characterization of EVT Transcriptome

#### Pathways Involved in EVT Differentiation and Maturation

To help characterize the differences between the largely diploid CTB and the majority polyploid EVT, we profiled the transcriptomes of isolated first trimester and term CTB and EVT, using RNA-seq. Our RNA-seq dataset was well powered, consisting of 10 first trimester CTB, 10 first trimester EVT, 10 term CTB, and 6 term EVT samples at an average depth of over 40 million uniquely mapped reads, offering a detailed look at how these two trophoblast cell types differed at two different GAs. With respect to first trimester EVT, our GSEA results confirmed previously published microarray datasets (Apps et al., 2011; Telugu et al., 2013; Tilburgs et al., 2015; Wakeland et al., 2017) and added further evidence that EVT differentiation entails upregulation of EMT and hypoxia signaling, along with many inflammatory- and immune-mediated processes, and eventually a downregulation of proliferation and cell cycle pathways suggesting terminal differentiation. Perhaps surprisingly, many of the same pathways were up- or down-regulated when comparing CTB and EVT from term placenta, suggesting that, despite a previous report (McMaster et al., 1995), given the right conditions, it may be possible to differentiate term CTB to EVT. Interestingly, two pathways that were found to be up-regulated in first trimester EVT and not term EVT were the PI3K/AKT/mTOR signaling and UPR pathways. By inhibiting one arm of the UPR pathway during EVT differentiation of primary hTSC, we found that this pathway is important for surface expression of HLA-G, a molecule required for EVT crosstalk with maternal natural killer (NK) cells (Pollheimer et al., 2018). ER stress has been studied in the context of oxidative stress-induced placental dysfunction (i.e., in the setting of pre-eclampsia and intrauterine growth restriction; Burton et al., 2009; Mizuuchi et al., 2016). It is also known that enhanced induction of this pathway disrupts placental development (Yung et al., 2012), but, until now, it had not been studied specifically in the context of EVT. Interestingly, hypoxic conditions, known to promote EVT differentiation (Wakeland et al., 2017), induce adaptive cellular responses including the UPR pathway. It is tempting to speculate that hypoxia-induced EVT differentiation is partially mediated through the IRE1-alpha arm of UPR; further studies are needed to test this hypothesis.

At the same time, we also noted a large overlap in pathways that were significantly different in first trimester CTB vs. EVT and first trimester vs. term EVT. This suggests that these overlapping pathways may be essential for both differentiation and maturation of, or simply characteristic of both immature and mature, EVT. Many such pathways are likely to involve signals from the decidua and decidual immune cells, of which NK and macrophages are the most abundant (Vento-Tormo et al., 2018; Pique-Regi et al., 2019). Given the lack of polyploidy in our *in vitro*-differentiated EVT, it is worth exploring which, if any, of these pathways require further manipulation in order to optimize EVT differentiation of hTSC lines *in vitro*.

Finally, we evaluated gene expression differences between male and female EVTs and identified a relatively small number of DEGs between these groups; interestingly, the number of DEGs was higher at term (51) compared with first trimester (16). Although these DEGs were not enriched in any specific pathways, the findings correlate with those of Papuchova et al. (2020), showing that term (but not first trimester) EVTs showed higher levels of HLA-G if they came from placenta of a male fetus. Given this, and the well-known role of sexual dimorphism in placental development and disease (Kalisch-Smith et al., 2017), further study of these DEGs in EVT function is warranted.

#### Gene Expression Changes Associated With Polyploidy and Senescence

Polyploidy has long been intricately linked with cellular senescence (Campisi and d’Adda di Fagagna, 2007), and a recent report has suggested that first trimester EVTs exhibit both endoreduplication-induced polyploidy and senescence (Velicky et al., 2018). Therefore, we examined our RNA-seq data for genes involved in the cell cycle, endoreduplication, and cellular senescence. We found that term EVT did not express mitosis-linked genes, such as cyclin B, Ki67, and Aurora kinase A, but all three genes were expressed in first trimester EVT, in contrast to previous reports (Velicky et al., 2018). As discussed above, endoreduplication consists of DNA replication without cell or nuclear division and is triggered by p57 (CDKN1C) inhibition of CDK1 and p21 (CDKN1A) suppression of CHEK1, preventing induction of apoptosis (Ullah et al., 2008). Our term EVT showed marked decrease in CDK1 and CHEK1 and corresponding increases in p57 and p21, suggesting that, as in mouse TGCs, human term EVTs undergo endoreduplication. Interestingly, the G1–S transition promoting cyclin E1 was highly expressed in first trimester EVT, but nearly 3-fold lower in term EVT, whereas cyclin E2 progressively decreased between both CTB, first trimester EVT, and term EVT. Additionally, the mitosis-linked cyclin B gene was highly expressed in first trimester EVT but almost undetectable in term EVT. However, despite Velicky et al.’s claim that Cyclin A^+^/p57^–^ expression could be used as a marker for endoreduplicating HLA-G^+^ trophoblast, we found very low levels of cyclin A in all of our samples despite relatively deep sequencing. We did observe that the genes encoding the RB protein and its E2F TF family targets also drop precipitously in term EVT, whereas SASP-associated genes were most highly expressed in term EVT. In the context of our FISH and flow cytometry data, these results suggest that cells may begin to undergo endoreduplication and senescence in the first trimester, but progress into a more fully senescent phenotype only at term. However, further studies, including evaluation of protein and phosphorylation levels (including RB protein phosphorylation; Giacinti and Giordano, 2006), should be conducted to more precisely evaluate the cell cycle during EVT formation.

It should be noted that, while Velicky et al. defined EVT spatially (as cells within the distal portion of the trophoblast cell column with larger nuclei), we defined EVT in this study based on surface HLA-G expression. Within first trimester samples, this likely led to a more heterogenous mixture of EVT in our samples, containing more proximal cell column trophoblasts, which are more proliferative and less mature, leading to some of the discrepancies between our two studies. More mature EVTs are found within decidual tissue strips (in first trimester samples) and deeper within the placental bed (in term samples). Though more difficult to obtain, future studies should attempt to include such samples and thus more thoroughly assess processes involved in EVT maturation.

#### TF Networks Regulating EVT Differentiation and Maturation

Using our RNA-seq data, we sought to identify TFs needed to drive EVT differentiation and maturation. We found that many TFs identified as being important in our protein-to-protein interaction networks were well-known regulators of processes previously identified as being vital in normal EVT development. During differentiation of first trimester CTB to EVT, we found the top four TFs with the highest importance scores in our largest clustered subnetwork to be TFEB, IRF7, IRF8, and STAT1, all of which are involved in immune response (Martina et al., 2012; Brady et al., 2018; Jefferies, 2019; Jung et al., 2020), correlating well with our GSEA findings (Zhang et al., 2015). We validated STAT1 as uniquely expressed in EVT within first trimester placental tissue, with its expression increasing during *in vitro* differentiation of hTSC into EVT; similar findings were recently reported by Chen et al. (2021), who have suggested that the signals leading to STAT1 induction are derived from decidual stromal cells. Additionally, we noted that this clustered subnetwork, and the largest subnetwork in the genes up-regulated in term EVT vs. CTB, was centered around FN1, which, we found to be linked with XBP1, a key part of the UPR pathway in mammals (Hetz and Papa, 2018). XBP1 was recently reported to initiate FN1 expression in colon cancer cells (Xie et al., 2019) and thus deserves further study as a possible link between hypoxia, UPR, and ECM remodeling in the context of EVT differentiation.

In our analysis of TFs involved in EVT maturation, we identified GCM1 as the TF with the highest importance score in the largest subnetwork of genes up-regulated in term, compared with first trimester, EVT, though its expression appeared to decrease in term, compared with first trimester, EVT; we validated these findings by ISH, using both first trimester and term placental tissues. GCM1 is a TF known best as a master regulator of labyrinthine or villous trophoblast differentiation in both mice and human (Cross et al., 2006; Baczyk et al., 2009). However, we and others have shown that GCM1 is also highly expressed in human EVT (Baczyk et al., 2004; Chiu and Chen, 2016; Wakeland et al., 2017). GCM1 has been shown to play a role in trophoblast invasion, acting through HTRA4, a serine protease that facilitates fibronectin cleavage, to suppress cell–cell fusion and promote invasion (Wang et al., 2012). However, while this describes a clear role for GCM1 in a basic function (invasion) of all EVT, including those in first trimester, its identification as a key TF involved in regulation of term EVT transcriptome requires further study.

Other TFs identified as controlling up-regulated genes during EVT maturation included NFAT5 and STAT4, both of which are involved in immune response, with the latter regulating response to IL-12 signaling (Morinobu et al., 2002; Lee et al., 2019). The IL-12 cytokine family, produced by EVT, is important in establishment of maternal–fetal tolerance through modulation of naïve conventional T cells and their conversion into induced regulatory T cells (Liu et al., 2019). Papuchova et al. (2020) has pointed to heterogeneity within term EVT, with subtypes showing differing capacities for modulating resident immune cells, including regulatory T cells. Future studies, including single-cell analysis, are warranted to further study EVT heterogeneity, based not just on ploidy and gene expression, but on functional capacities, in order to better understand the role of these cells in establishment and maintenance of the maternal–fetal interface.

### Summary

In summary, our study builds on earlier reports characterizing first trimester EVT, extending such genomic and transcriptomic studies to term EVT, and defining pathways and TF networks involved in both initial differentiation and maturation of this important trophoblast lineage at the maternal–fetal interface. Our results suggest that term EVTs lack high rates of CNVs, though studies using WGS with substantially larger sample sizes are needed to definitively identify or rule-out the presence of functionally relevant under- or over-represented genomic regions. Additionally, we have highlighted senescence and polyploidy-related genes, pathways, networks, and TFs that appeared to be important in EVT differentiation and maturation and have validated a critical role for the UPR in formation of functional EVT. Lastly, our results highlight the need for more optimized *in vitro* models of EVT differentiation, further research into functional differences among EVT subpopulations with different ploidy levels, and studies of placental diseases that may be associated with changes in cellular ploidy or dysfunctional EVT differentiation or maturation.

## Data Availability Statement

The datasets presented in this study can be found in online repositories. The names of the repository/repositories and accession number(s) can be found below: BioProject, accession: PRJNA724881, GEO accession: GSE173372 https://www.ncbi.nlm.nih.gov/geo/query/acc.cgi?acc=GSE173372.

## Ethics Statement

The studies involving human participants were reviewed and approved by Human Research Protections Program Committee of the UCSD Institutional Review Board (IRB number: 181917X). The patients/participants provided their written informed consent to participate in this study. Written informed consent was obtained from the individual(s) for the publication of any potentially identifiable images or data included in this article.

## Author Contributions

RM, OF, DR, SK, FS, and MM performed the experiments. RM, OF, DR, and SK performed the data analysis. LL and MP supervised and designed the study. RM, LL, and MP wrote the manuscript. All authors contributed to the article and approved the submitted version.

## Conflict of Interest

The authors declare that the research was conducted in the absence of any commercial or financial relationships that could be construed as a potential conflict of interest.

## Publisher’s Note

All claims expressed in this article are solely those of the authors and do not necessarily represent those of their affiliated organizations, or those of the publisher, the editors and the reviewers. Any product that may be evaluated in this article, or claim that may be made by its manufacturer, is not guaranteed or endorsed by the publisher.

## Footnotes

1
https://genomics.senescence.info/download.html#cellage

## Supplementary Material

Supplementary Figure 1 Ploidy determination of whole genome sequencing data and SNP genotyping array data. **(A)** Ploidy of two HLAG^+^ samples (11- and 12-weeks gestational age) from BioProject accession PRJNA445189 (Velicky et al., 2018) as determined by PURPLE reanalysis. **(B)** Ploidy of SNP genotyping array of our term EVT samples as determined by ASCAT.

Supplementary Figure 2 Differential gene expression between male and female samples in first trimester and term EVT. **(A)** Heatmap of all differentially expressed genes between male and female samples in first trimester EVT. Blue- and pink- highlighted dendrogram rows are genes up-regulated in male or female samples, respectively. **(B)** Heatmaps of differentially expressed genes between male and female samples in term EVT, with genes up-regulated in the female samples shown in top heatmap and those up-regulated in male samples in bottom heatmap. For both analyses, differentially expressed genes were determined by adj *p*-value < 0.05, Log_2_ fold change > 1, mean normalized expression in group > 100; values were log transformed to create heatmap.

Supplementary Figure 3 Common and unique pathways involved in EVT differentiation and maturation. **(A)** GSEA using only founder gene sets of the two common down-regulated pathways during EVT maturation (first trimester EVT → term EVT; E2F targets and G2M checkpoint pathways) showed down-regulation in term EVT for the neighborhood of CCNA2 (Cyclin A2), PCNA, and RRM2 in the GNF2 expression compendium. The three genes shown in the figure are representative genes from each of these gene sets. **(B)** GSEA enrichment score plot showing Hallmark pathway Unfolded Protein Response (UPR) gene set in first trimester EVT compared to term EVT. The UPR pathway is also enriched in first trimester EVT compared to first trimester CTB.

Supplementary Figure 4 Principal component analysis of RNA-seq data comparing our three hTSC lines and those previously reported (Okae et al., 2018). Principal component analysis shows the first two components using all common genes between the two datasets post-filtering. The plot shows all 36 placenta samples from this study and 6 from Okae et al. (2018), triplicates of three hTSC lines (1,048, 1,049, and 1,270) from this study, and duplicates of blastocyst derived hTSC and placental derived hTSC from Okae et al. (2018; see Supplementary Table 1).

Supplementary Figure 5 The role of IRE1-alpha arm of the Unfolded Protein Response (UPR) pathway in EVT differentiation of hTSCs. Two different hTSC lines (1,048 and 1,049) were differentiated into EVT over 5 days in the presence or absence of the IRE1-alpha inhibitor, 4u8C. **(A)** qPCR for markers of CTB (ITGA6) or EVT (ITGA5 and ITGA1). **(B)** qPCR for markers of EVT (HLAG and ASCL2). **(C)** qPCR for UPR pathway genes, XBP1 (total and spliced) and ATF4. The decrease in spliced XBP1 following 4u8C treatment confirms inhibition of the IRE1-alpha arm of UPR. ddCT values were normalized to beta-actin and shown as fold change over day 0. ^∗^ shows statistically significant difference from day 0, while ^#^ shows statistically significant difference from DMSO carrier alone treatment on the same day, based on *t*-test (*p* < 0.05).

Supplementary Figure 6 Expression of cell cycle-associated genes in first trimester and term CTB and EVT. **(A)** Normalized gene expression in all samples for AURKA, CDK1, MKI67, and CCNB1. **(B)** Cell cycle scoring based on previously-identified cell cycle phase-specific gene expression (Macosko et al., 2015) on term single-cell RNA-seq data (Tsang et al., 2017) visualized in a UMAP representation. HLAG and EGFR expression are shown to identify EVT and CTB cell clusters, respectively. **(C)** Cell cycle and endoreduplication-associated gene expression (normalized counts) of Cyclin E1 (CCNE1).

Supplementary Table 1 Details of all samples used in study. CTB, cytotrophoblast; EVT, extravillous trophoblast; MSC, umbilical cord-derived mesenchymal stem cells; and TSC, trophoblast stem cells (derived from first trimester placentae).

Supplementary Table 2 List of primers used for qPCR.
