# Corrigendum: Transcriptomic drivers of differentiation, maturation, and polyploidy in human extravillous trophoblast

**DOI:** 10.3389/fcell.2023.1189745

**Published:** 2023-03-28

**Authors:** Robert Morey, Omar Farah, Sampada Kallol, Daniela F. Requena, Morgan Meads, Matteo Moretto-Zita, Francesca Soncin, Louise C. Laurent, Mana M. Parast

**Affiliations:** ^1^ Department of Pathology, University of California, San Diego, La Jolla, CA, United States; ^2^ Department of Obstetrics, Gynecology, and Reproductive Sciences, Division of Maternal-Fetal Medicine, University of California, San Diego, La Jolla, CA, United States; ^3^ Sanford Consortium for Regenerative Medicine, University of California, San Diego, La Jolla, CA, United States

**Keywords:** extravillous trophoblast, placenta, polyploid, senescence, cytotrophoblast

In the published article, there was an error in Supplementary Table S1. The fetal sex for patient number 1049 was displayed as “Male”. The correct material statement appears below.

The correct fetal sex for patient number 1049 is “Female”.

The authors apologize for this error and state that this does not change the scientific conclusions of the article in any way. The original article has been updated.

