# Supplementary figures and images for: Transcriptomic Drivers of Differentiation, Maturation, and Polyploidy in Human Extravillous Trophoblast
Source: Front Cell Dev Biol. 2021 Sep 3;9:702046. doi: 10.3389/fcell.2021.702046 (PMC8446284; doi:10.3389/fcell.2021.702046)

A

Ploidy of Two HLA-G+ Human WGS Samples Determined by PURPLE

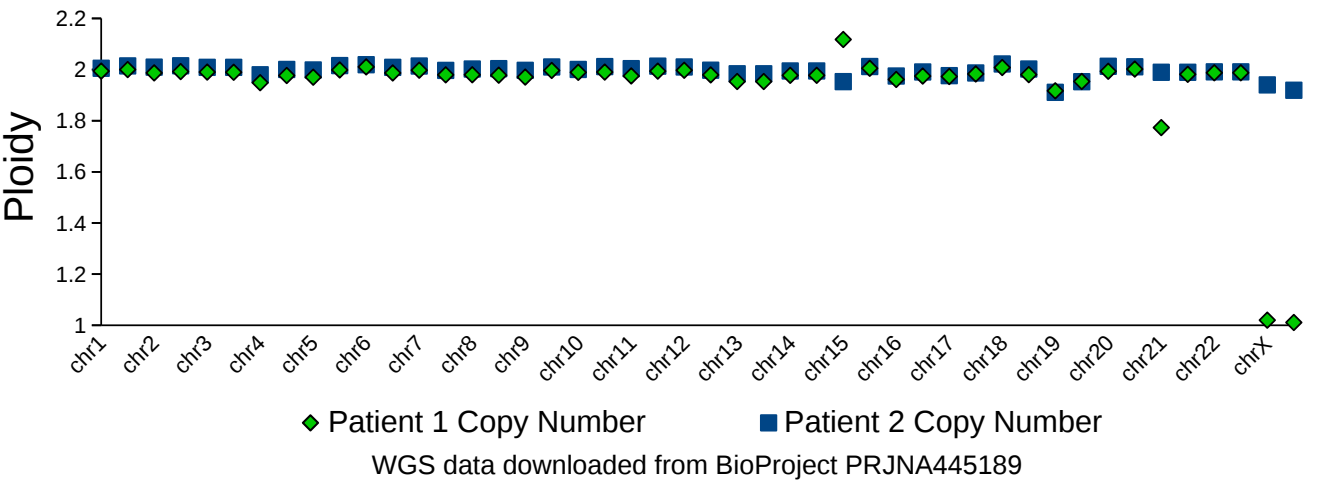

B

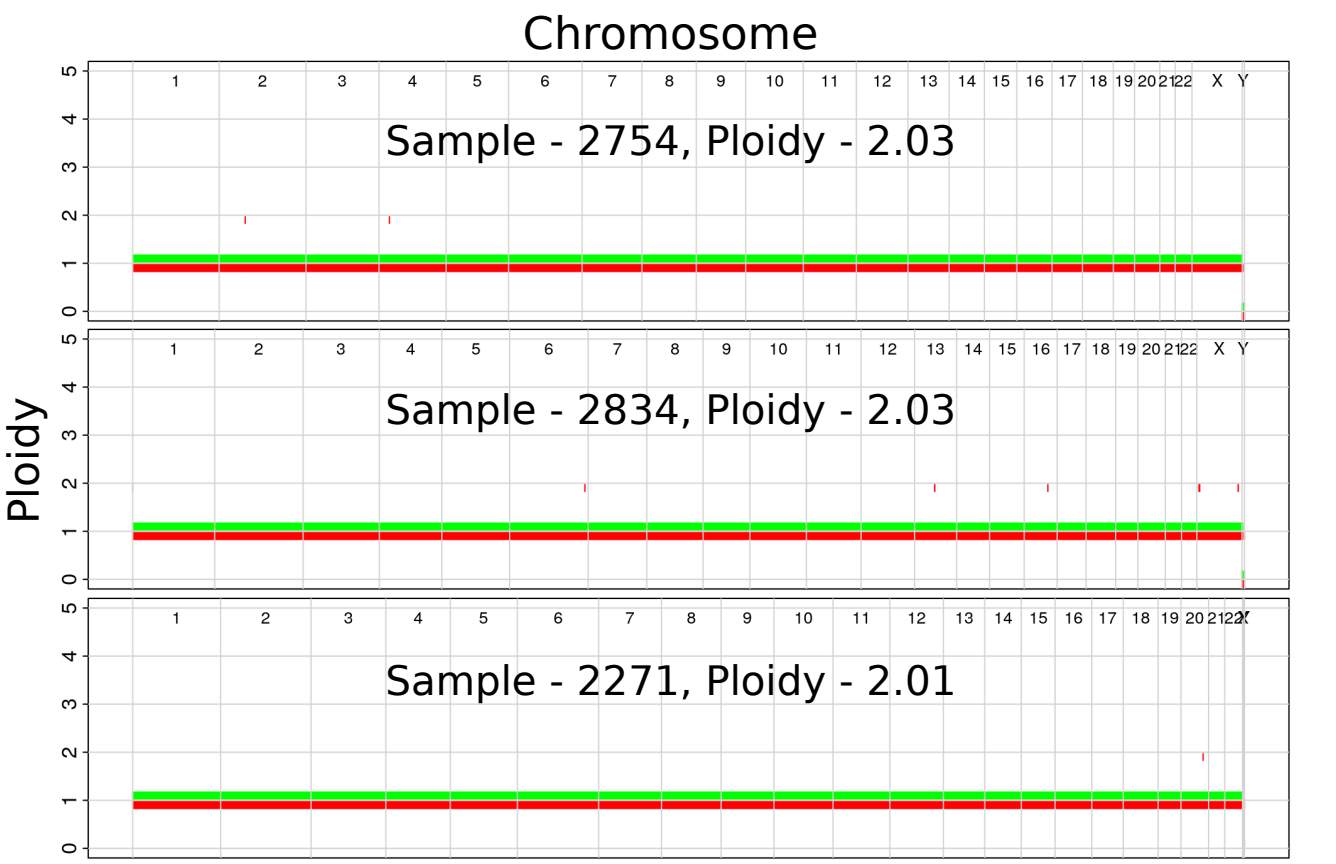

Supplement: Supplementary Figure 1 — Ploidy determination of whole genome sequencing data and SNP genotyping array data. (A) Ploidy of two HLAG+ samples (11- and 12-weeks gestational age) from BioProject accession PRJNA445189 (Velicky et al., 2018) as determined by PURPLE reanalysis. (B) Ploidy of SNP genotyping array of our term EVT samples as determined by ASCAT. [file Data_Sheet_1.PDF]

# A

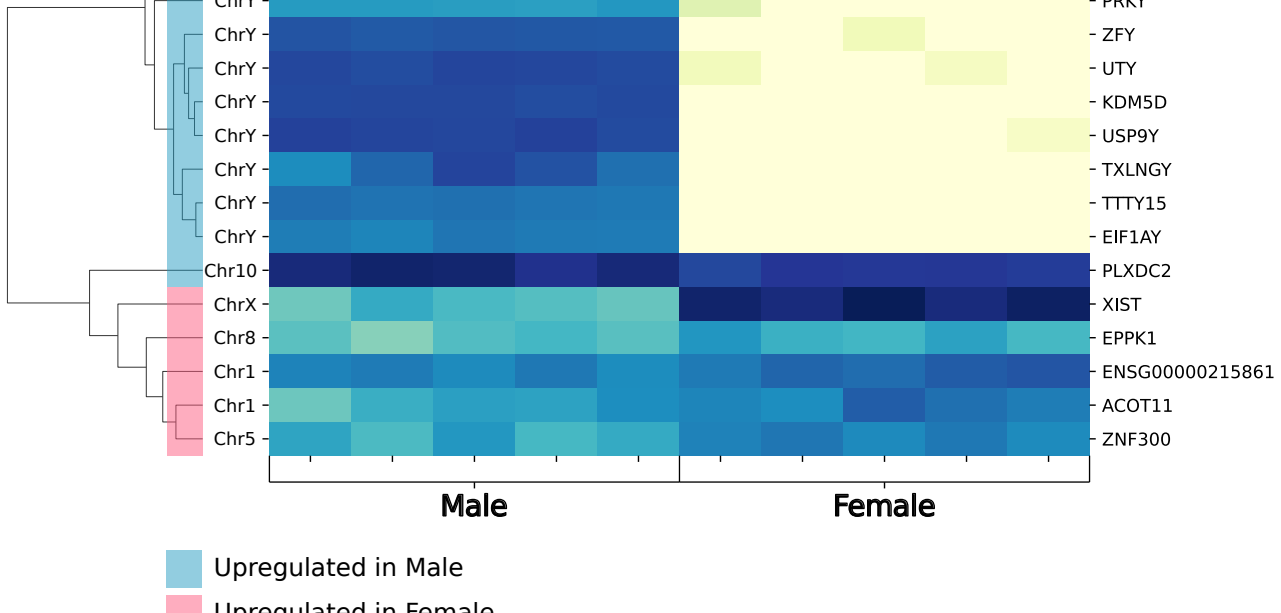

# B

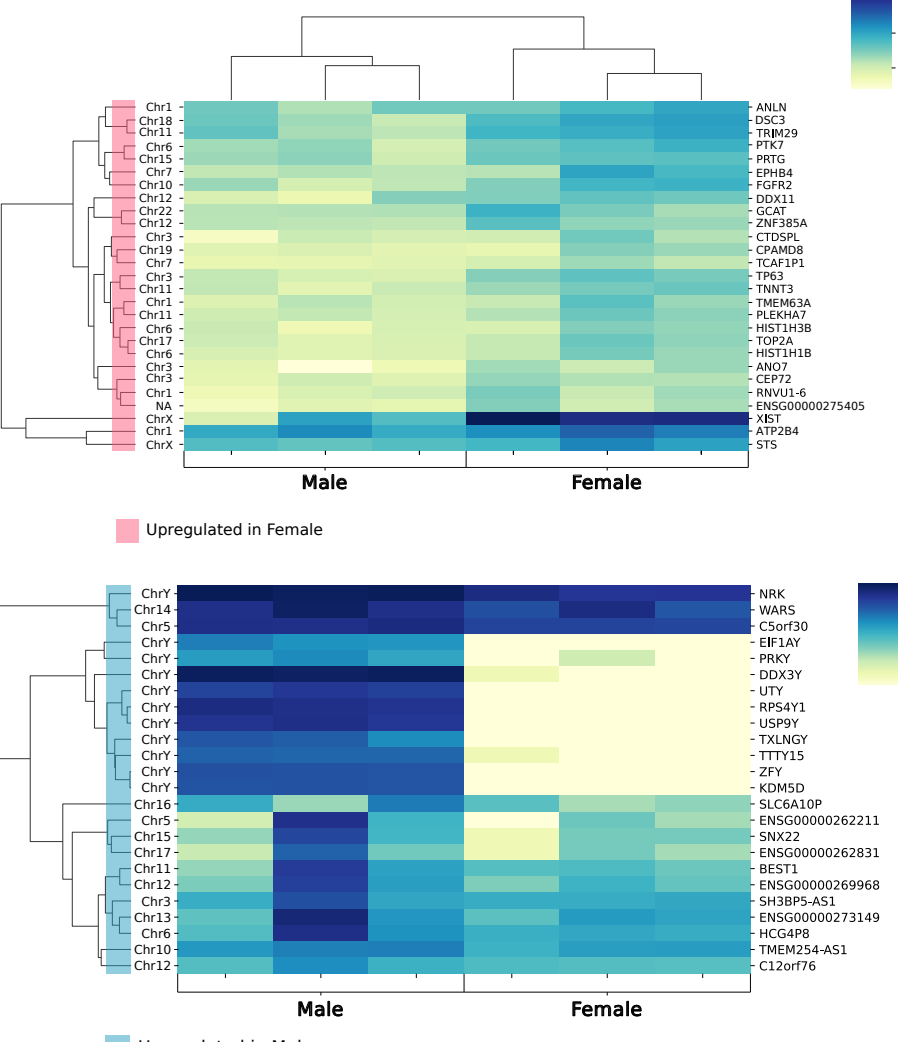

Supplement: Supplementary Figure 2 — Differential gene expression between male and female samples in first trimester and term EVT. (A) Heatmap of all differentially expressed genes between male and female samples in first trimester EVT. Blue- and pink- highlighted dendrogram rows are genes up-regulated in male or female samples, respectively. (B) Heatmaps of differentially expressed genes between male and female samples in term EVT, with genes up-regulated in the female samples shown in top heatmap and those up-regulated in male samples in bottom heatmap. For both analyses, differentially expressed genes were determined by adj p-value < 0.05, Log2 fold change > 1, mean normalized expression in group > 100; values were log transformed to create heatmap. [file Data_Sheet_2.PDF]

A

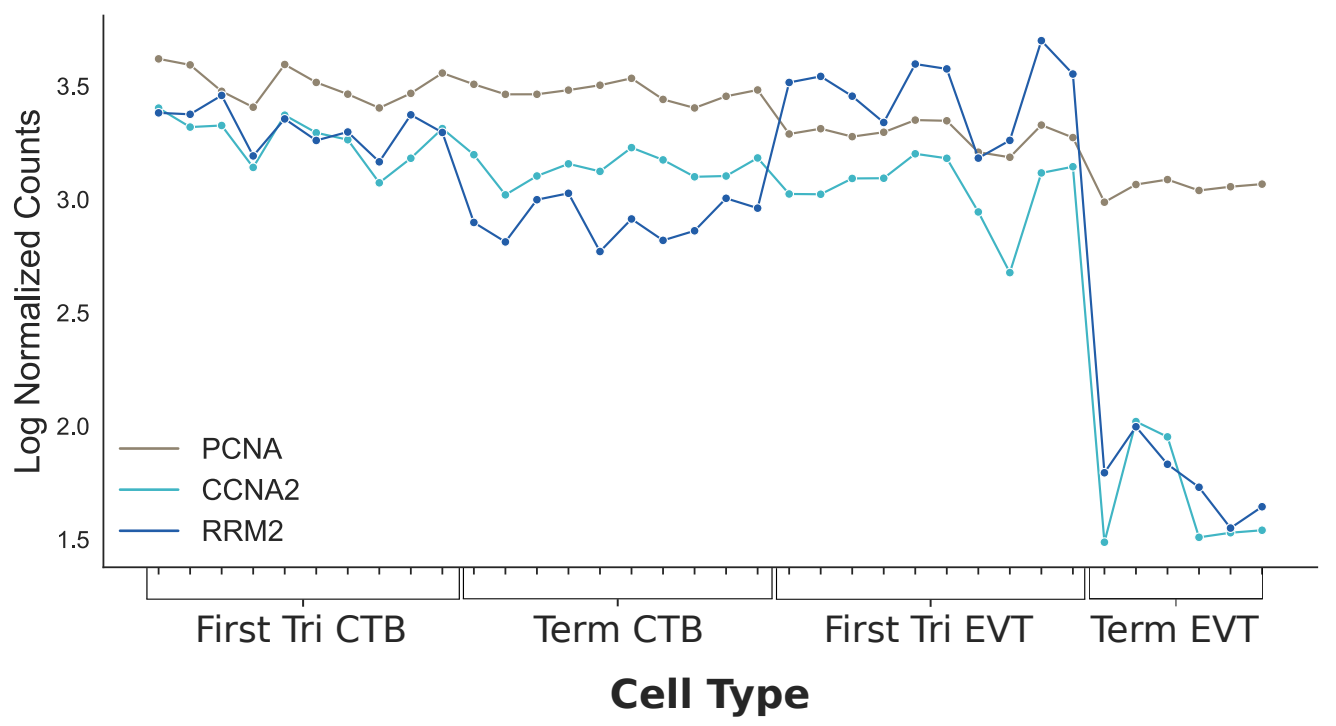

B

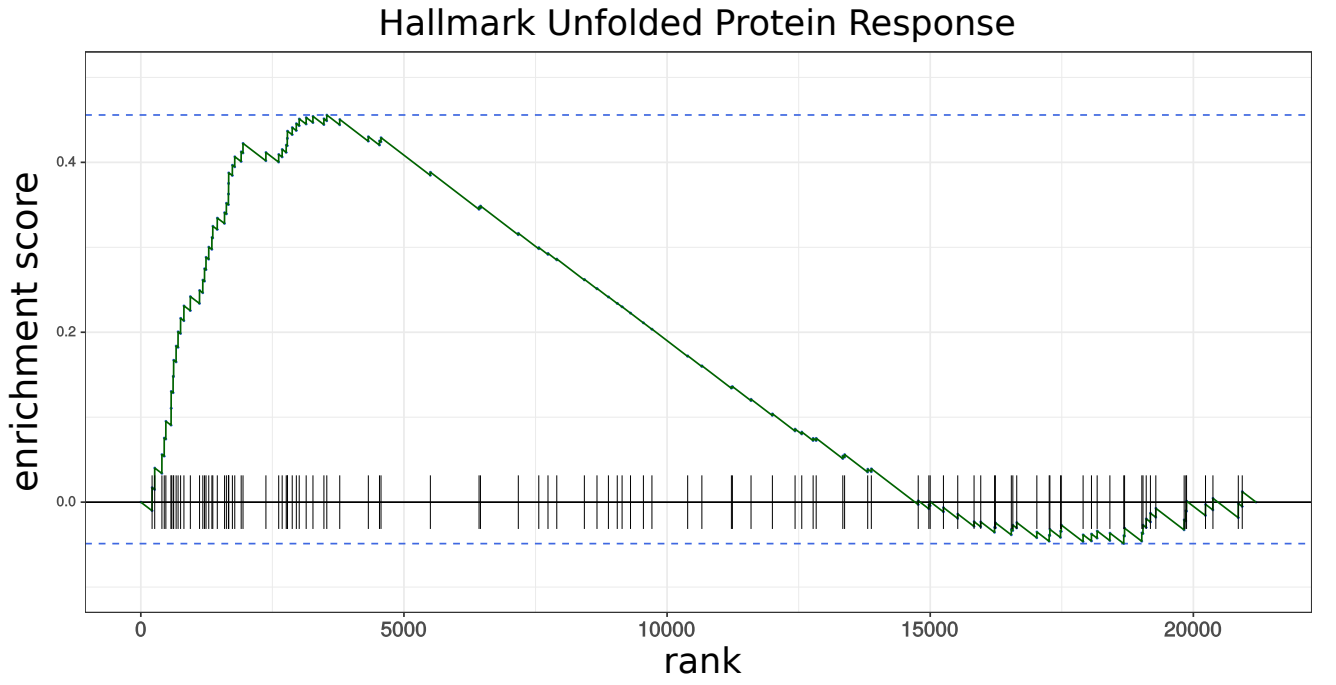

Supplement: Supplementary Figure 3 — Common and unique pathways involved in EVT differentiation and maturation. (A) GSEA using only founder gene sets of the two common down-regulated pathways during EVT maturation (first trimester EVT → term EVT; E2F targets and G2M checkpoint pathways) showed down-regulation in term EVT for the neighborhood of CCNA2 (Cyclin A2), PCNA, and RRM2 in the GNF2 expression compendium. The three genes shown in the figure are representative genes from each of these gene sets. (B) GSEA enrichment score plot showing Hallmark pathway Unfolded Protein Response (UPR) gene set in first trimester EVT compared to term EVT. The UPR pathway is also enriched in first trimester EVT compared to first trimester CTB. [file Data_Sheet_3.PDF]

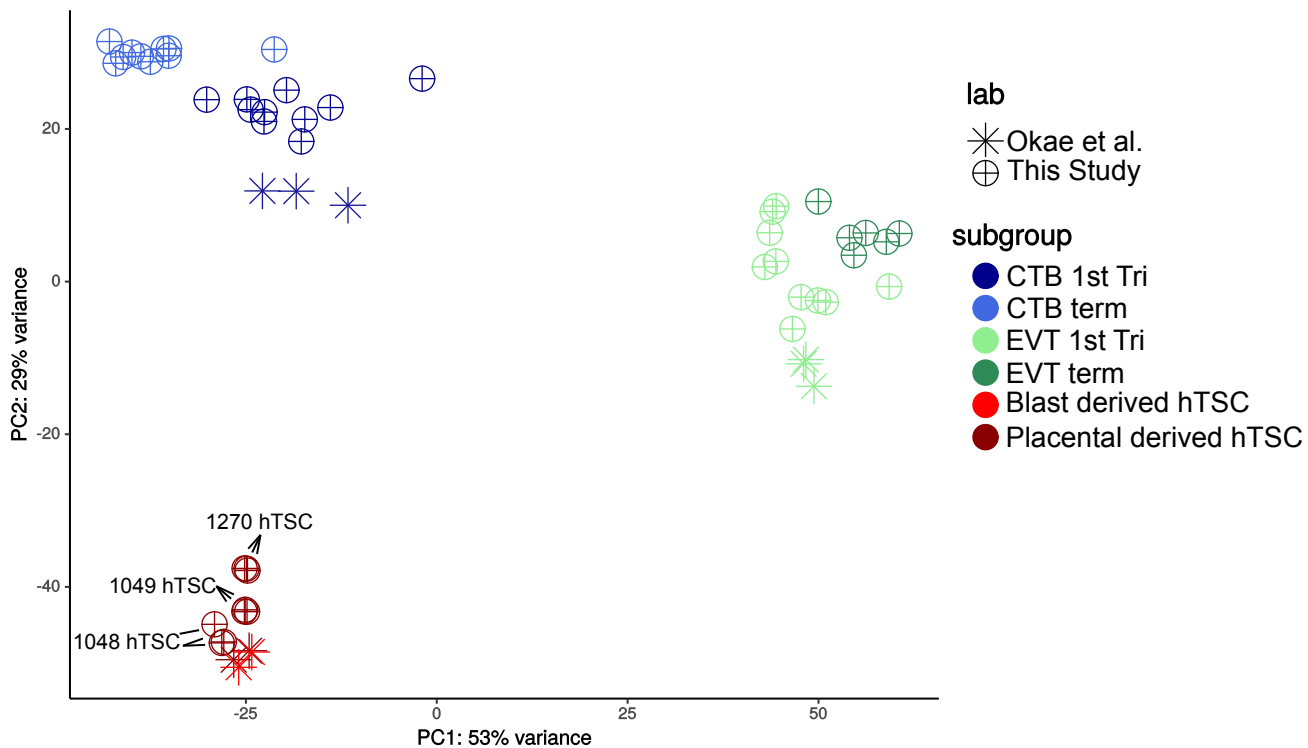

Supplement: Supplementary Figure 4 — Principal component analysis of RNA-seq data comparing our three hTSC lines and those previously reported (Okae et al., 2018). Principal component analysis shows the first two components using all common genes between the two datasets post-filtering. The plot shows all 36 placenta samples from this study and 6 from Okae et al. (2018), triplicates of three hTSC lines (1,048, 1,049, and 1,270) from this study, and duplicates of blastocyst derived hTSC and placental derived hTSC from Okae et al. (2018; see Supplementary Table 1). [file Data_Sheet_4.PDF]

A

1048 TSC-EVT markers

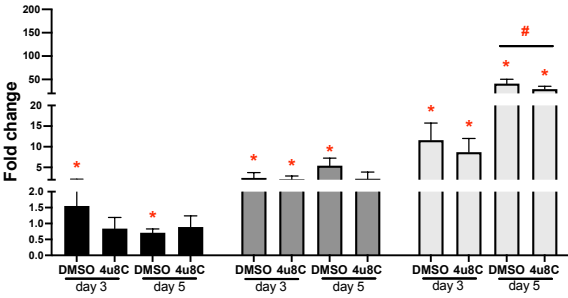

1049 TSC-EVT markers

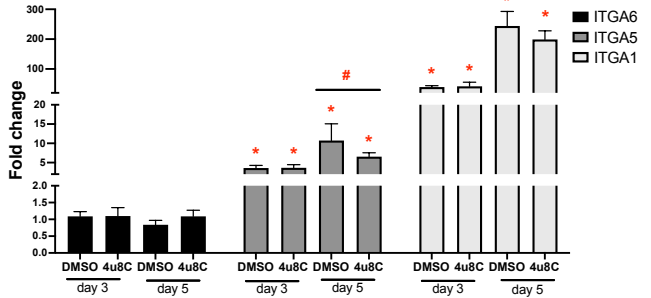

B

1048 EVT markers

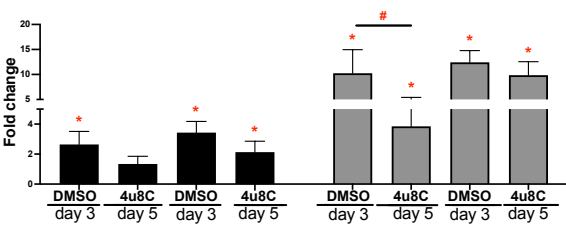

1049 EVT markers

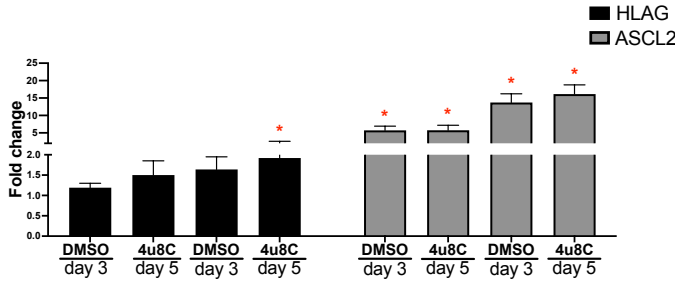

C

1048 ER stress markers

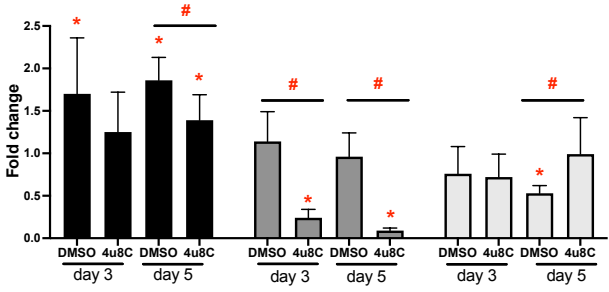

1049 ER stress markers

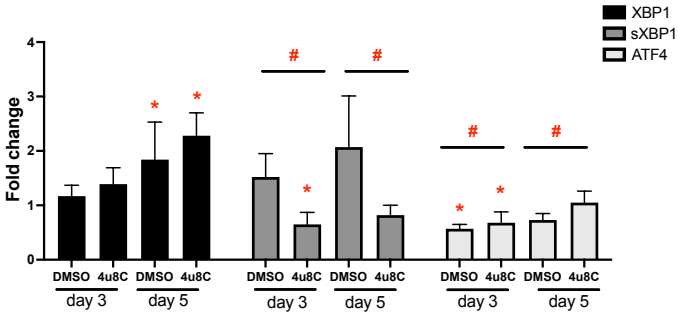

Supplement: Supplementary Figure 5 — The role of IRE1-alpha arm of the Unfolded Protein Response (UPR) pathway in EVT differentiation of hTSCs. Two different hTSC lines (1,048 and 1,049) were differentiated into EVT over 5 days in the presence or absence of the IRE1-alpha inhibitor, 4u8C. (A) qPCR for markers of CTB (ITGA6) or EVT (ITGA5 and ITGA1). (B) qPCR for markers of EVT (HLAG and ASCL2). (C) qPCR for UPR pathway genes, XBP1 (total and spliced) and ATF4. The decrease in spliced XBP1 following 4u8C treatment confirms inhibition of the IRE1-alpha arm of UPR. ddCT values were normalized to beta-actin and shown as fold change over day 0. ∗ shows statistically significant difference from day 0, while # shows statistically significant difference from DMSO carrier alone treatment on the same day, based on t-test (p < 0.05). [file Data_Sheet_5.PDF]

A

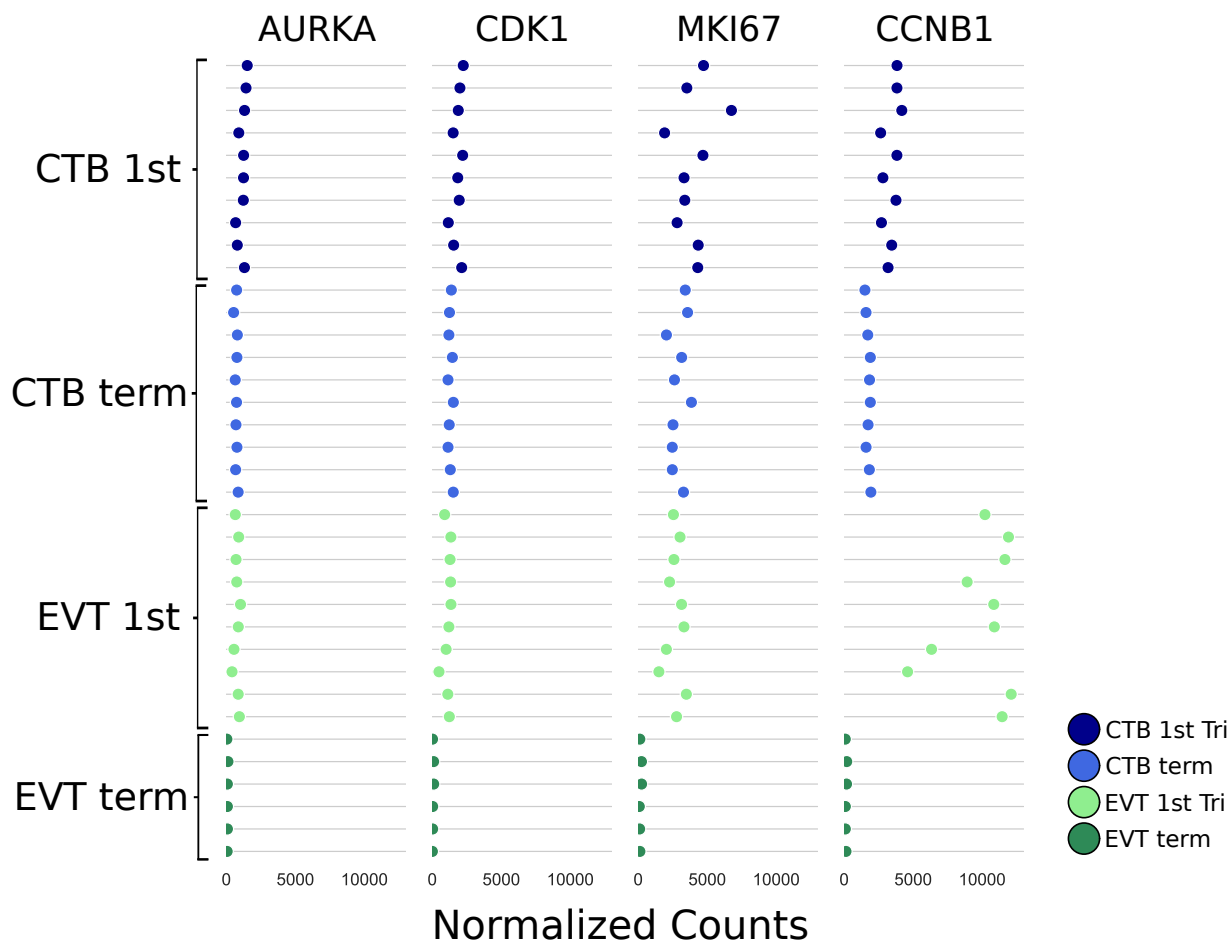

B

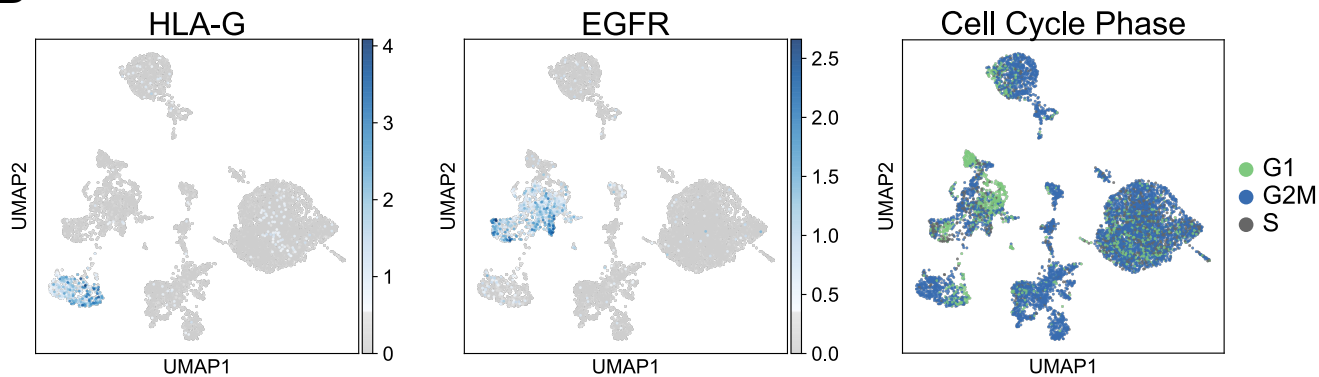

C

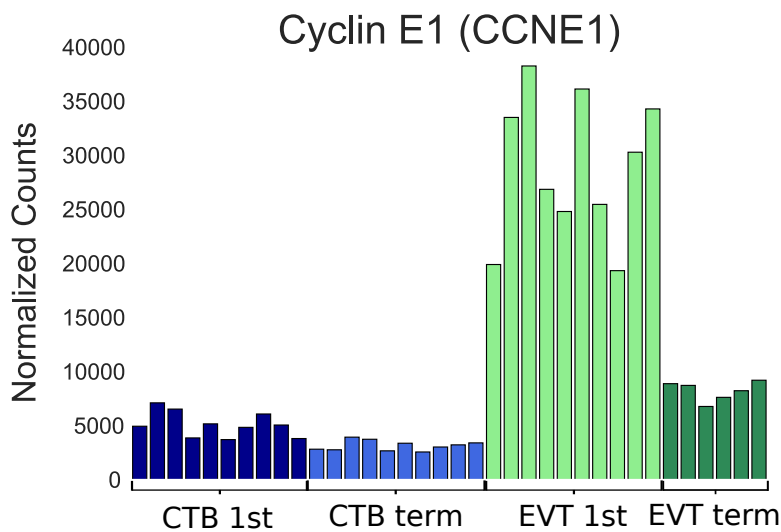

Supplement: Supplementary Figure 6 — Expression of cell cycle-associated genes in first trimester and term CTB and EVT. (A) Normalized gene expression in all samples for AURKA, CDK1, MKI67, and CCNB1. (B) Cell cycle scoring based on previously-identified cell cycle phase-specific gene expression (Macosko et al., 2015) on term single-cell RNA-seq data (Tsang et al., 2017) visualized in a UMAP representation. HLAG and EGFR expression are shown to identify EVT and CTB cell clusters, respectively. (C) Cell cycle and endoreduplication-associated gene expression (normalized counts) of Cyclin E1 (CCNE1). [file Data_Sheet_6.PDF]
