# Supplementary material for: Transcriptomic Drivers of Differentiation, Maturation, and Polyploidy in Human Extravillous Trophoblast
Source: Front Cell Dev Biol. 2021 Sep 3;9:702046. doi: 10.3389/fcell.2021.702046 (PMC8446284; doi:10.3389/fcell.2021.702046)
Supplement: Supplementary Table 2 — List of primers used for qPCR. [file Table_2.DOCX]

Supplementary Table 2: List of qRT-PCR primers

| **Gene name** | **Primer pairs (5’- end to 3’ - end)** |
| --- | --- |
| ACTB (beta actin) | for: CGCACCACTGGCATTGTCAT  rev: TTCTCCTTGATGTCACGCAC |
| ASCL2 | for: CACTGCTGGCAAACGGAGAC  rev: AAAACTCCAGATAGTGGGGGC |
| ATF4 | for: GTTCTCCAGCGACAAGGCTA  rev: ATCCTGCTTGCTGTTGTTGG |
| EGFR | for: CTAAGATCCCGTCCATCGCC  rev: GGAGCCCAGCACTTTGATCT |
| HLAG | for: ACTGAGTGGCAAGTCCCTTT  rev: TGGGGAAGGAATGCAGTTCAG |
| ITGA1 | for: CTGGACATAGTCATAGTGCTGGA  rev: ACCTGTGTCTGTTTAGGACCA |
| ITGA5 | for: GGCTTCAACTTAGACGCGGAG  rev: TGGCTGGTATTAGCCTTGGGT |
| ITGA6 | for: GGCGGTGTTATGTCCTGAGTC  rev: AATCGCCCATCACAAAAGCTC |
| p63 | for: CTGGAAAACAATGCCCAGA  rev: AGAGAGCATCGAAGGTGGAG |
| sXBP1 | for: CTGAGTCCGAATCAGGTGCAG  rev: ATCCATGGGGAGATGTTCTGG |
| XBP1 | for: TGGCCGGGTCTGCTGAGTCCG  rev: ATCCATGGGGAGATGTTCTGG |
